# Pain and its interference with daily living in relation to cancer: a comparative population-based study of 16,053 cancer survivors and 106,345 people without cancer

**DOI:** 10.1186/s12885-023-11214-5

**Published:** 2023-09-13

**Authors:** Grace Joshy, Saman Khalatbari-Soltani, Kay Soga, Phyllis Butow, Rebekah Laidsaar-Powell, Bogda Koczwara, Nicole M. Rankin, Sinan Brown, Marianne Weber, Carolyn Mazariego, Paul Grogan, John Stubbs, Stefan Thottunkal, Karen Canfell, Fiona M. Blyth, Emily Banks

**Affiliations:** 1 National Centre for Epidemiology and Population Health (NCEPH), The Australian National University, Canberra, ACT Australia; 2 Sydney School of Public Health, The University of Sydney, Sydney, NSW Australia; 3 Centre for Medical Psychology and Evidence-Based Decision-Making (CeMPED), School of Psychology, The University of Sydney, Sydney, NSW Australia; 4 Flinders Health and Medical Research Institute, Flinders University, Adelaide, SA Australia; 5 Department of Medical Oncology, Flinders Medical Centre, Adelaide, SA Australia; 6 Melbourne School of Population and Global Health, The University of Melbourne, Melbourne, VIC Australia; 7 The Daffodil Centre, The University of Sydney, a joint venture with Cancer Council NSW, Sydney, NSW Australia; 8 School of Population Health, The University of New South Wales, Sydney, NSW Australia; 9 Independent Cancer Consumer Advisor, Sydney, NSW Australia

**Keywords:** Cancer, Pain, Pain interference, High-impact pain, Physical functioning, Psychological distress, Quality of life

## Abstract

**Background:**

Pain is a common, debilitating, and feared symptom, including among cancer survivors. However, large-scale population-based evidence on pain and its impact in cancer survivors is limited. We quantified the prevalence of pain in community-dwelling people with and without cancer, and its relation to physical functioning, psychological distress, and quality of life (QoL).

**Methods:**

Questionnaire data from participants in the 45 and Up Study (Wave 2, *n* = 122,398, 2012–2015, mean age = 60.8 years), an Australian population-based cohort study, were linked to cancer registration data to ascertain prior cancer diagnoses. Modified Poisson regression estimated age- and sex-adjusted prevalence ratios (PRs) for bodily pain and pain sufficient to interfere with daily activities (high-impact pain) in people with versus without cancer, for 13 cancer types, overall and according to clinical, personal, and health characteristics. The relation of high-impact pain to physical and mental health outcomes was quantified in people with and without cancer.

**Results:**

Overall, 34.9% (5,436/15,570) of cancer survivors and 31.3% (32,471/103,604) of participants without cancer reported bodily pain (PR = 1.07 [95% CI = 1.05–1.10]), and 15.9% (2,468/15,550) versus 13.1% (13,573/103,623), respectively, reported high-impact pain (PR = 1.13 [1.09–1.18]). Pain was greater with more recent cancer diagnosis, more advanced disease, and recent cancer treatment. High-impact pain varied by cancer type; compared to cancer-free participants, PRs were: 2.23 (1.71–2.90) for multiple myeloma; 1.87 (1.53–2.29) for lung cancer; 1.06 (0.98–1.16) for breast cancer; 1.05 (0.94–1.17) for colorectal cancer; 1.04 (0.96–1.13) for prostate cancer; and 1.02 (0.92–1.12) for melanoma. Regardless of cancer diagnosis, high-impact pain was strongly related to impaired physical functioning, psychological distress, and reduced QoL.

**Conclusions:**

Pain is common, interfering with daily life in around one-in-eight older community-dwelling participants. Pain was elevated overall in cancer survivors, particularly for certain cancer types, around diagnosis and treatment, and with advanced disease. However, pain was comparable to population levels for many common cancers, including breast, prostate and colorectal cancer, and melanoma.

**Supplementary Information:**

The online version contains supplementary material available at 10.1186/s12885-023-11214-5.

## Background

Pain is distressing and debilitating and can profoundly impact quality of life (QoL), including the ability to work, move, and socialise [1, 2]. Globally, nearly 20% of adults suffer from pain [3]. Pain is one of the most common symptoms reported in people diagnosed with cancer [4]. Cancer survival is increasing in many higher income countries, including Australia, and the majority of people diagnosed with cancer now survive long-term, necessitating increased focus on survivor health and well-being [5]. Combined with demographic change, this means the number of individuals living with cancer is increasing rapidly and, in 2020, the five-year prevalence of all cancers worldwide (excluding non-melanoma skin cancer) led to estimates of over 44.1 million people affected [6].

Little is known about pain in community-dwelling cancer survivors and how it relates to other adverse outcomes, such as psychological distress and reduced QoL. Cancer is heterogeneous and evidence quantifying pain outcomes for different cancer types is also very limited, as is evidence on pain sufficient to interfere with work and home duties — as distinct from pain in general [7]. Most studies to date have involved small samples, single cancer types – mostly breast cancer – and/or lacked comparison to individuals without cancer. Of the 67 studies identified in the literature review (Additional file 1), 50, including the two studies based on Australian data, focused on assessment of change in the experience of pain over time in cancer survivors; they did not include a cancer-free control/comparison group. Only 17 studies included a comparison group of people without cancer, but they included little evidence for multiple cancer types or clinical characteristics. Previous studies have also varied in the measure of pain used, recall period, study design, characteristics of cancer survivors, and selection of controls. We could not find any comprehensive study on the relationship of cancer and pain, and the joint relationship of these to other adverse outcomes, such as psychological distress and reduced QoL.

We aimed to quantify the prevalence of pain and pain sufficient to impact on daily activities in community-dwelling people with and without a cancer diagnosis, and its relation to physical functioning limitations, psychological distress, QoL, and self-rated health. We investigated pain and high-impact pain overall, for 13 cancer types, and according to cancer stage, time since diagnosis, and recent treatment.

## Methods

### Study participants

The Sax Institute’s 45 and Up Study is a cohort study of 267,357 men and women aged 45 years or over, randomly sampled from the general population of New South Wales (NSW), Australia, using the Services Australia Medicare enrolment database. Regional and remote areas and those aged 80 years or over were oversampled; the study did not include people in residential aged care, community care facilities, or specialised geriatric services. Eligible community-dwelling individuals joined the study from 2005 to 2009 by completing postal questionnaires and consented to long-term follow-up through repeated surveys and linkage of their data to other population health databases [8]. This paper uses data from the Wave 2 follow-up survey, sent to participants between September 2012 and September 2015, and completed by 142,548 individuals [9].

Questionnaire data included comprehensive self-reported information including demographic factors, doctor-diagnosed health conditions, pain, functional capacity, self-rated health, and QoL [9]. Data from study participants were linked probabilistically to administrative datasets including the NSW Central Cancer Registry (CCR, 1 January 1994 to 31 December 2015) by the NSW Centre for Health Record Linkage; linkage is known to be highly accurate (false-positive and false-negative rates < 0.5%) [10]. The linked NSW CCR data comprised records of all diagnosed cancers including the date of diagnosis and International Statistical Classification of Diseases and Related Health Problems, Tenth Revision, Australian Modification (ICD-10-AM)-coded cancer types and sites, except basal cell carcinoma or squamous cell carcinoma (C44), which are not reported to the NSW CCR.

After excluding withdrawals (*n* = 136, 0.95%), those with invalid survey dates (*n* = 95, 0.07%) or survey dates in 2016 (i.e., after the cancer registry data cut-off date; *n* = 19,847, 13.9%), data linkage errors (*n* = 68, 0.056%), or baseline age under 45 years (*n* = 4, 0.003%), the analysis dataset consisted of 122,398 individuals.

### Exposures

The main exposure was a cancer diagnosis prior to completion of the follow-up questionnaire. Participants were considered to be cancer survivors if they had a cancer diagnosis record in the CCR database. The cancer type, time since diagnosis, and stage of cancer were also ascertained from the database. An 18-year look-back window, based on the availability of linked data, was used to ensure a uniform probability of identification of previous cancer diagnoses from the CCR database for all participants.

Thirteen common cancer types were selected based on incidence (worldwide and in Australia) [11, 12], case numbers in the study population, and known adverse impacts on well-being [13]. Cancers were classified as breast (women only), prostate (men only), lung, melanoma, colorectal, non-Hodgkin’s lymphoma (NHL), kidney, oesophagus, uterus (women only), bladder, thyroid, leukaemia, multiple myeloma, and ‘other cancers’ (Additional file 2: Table S1). If multiple cancers were present, the diagnosis closest to the study enrolment date was used.

Time since diagnosis was classified as < 1 year, 1- < 5 years, 5- < 10 years, and ≥ 10 years. The stage of cancer at diagnosis for solid tumours was classified as localised to the tissue of origin, regional spread to adjacent organs and/or regional lymph nodes, distant metastases, and unknown stage. Recent treatment was classified as ‘yes/no’ based on the response to the follow-up survey question, “In the last month, have you been treated for cancer?”. The reference group comprised respondents with no record of a cancer diagnosis in the CCR database prior to completion of the follow-up questionnaire.

### Outcomes

The main outcomes were based on validated measures of bodily pain and high-impact pain (pain sufficient to interfere with daily activities) reported on the follow-up survey [14]. Bodily pain was based on the question, “How much bodily pain have you had during the past 4 weeks?”, followed by response options of ‘none’, ‘very mild’, ‘mild’, ‘moderate’, ‘severe’, and ‘very severe’. Participants were considered to have bodily pain if they answered ‘moderate’, ‘severe’ or ‘very severe’. For high-impact pain, participants were asked, “During the past 4 weeks, how much did pain interfere with your normal work (including both work outside the home and housework)?”, followed by response options of ‘not at all’, ‘a little bit’, ‘quite a bit’, ‘moderately’, and ‘extremely’. In binary classification, participants were considered to have high-impact pain if they answered ‘moderately’ or ‘extremely’.

The relationship between the level of impact of pain and physical and mental health outcomes was investigated, including physical functioning limitations assessed using the Medical Outcomes Study Physical Functioning (MOS-PF) scale [15], psychological distress assessed using the Kessler 10 (K10) scale [16], self-rated health, and QoL. The MOS-PF is a valid and reliable measure of physical functioning [15], with a lower score indicating more severe functional limitation (scores can range from 0 to 100) [17]. The K10 is a validated measure of non-specific symptoms of psychological distress [16]. Respondents indicated the frequency of symptoms experienced in the past four weeks, from 1 ‘none of the time’ to 5 ‘all of the time’. Scores can range from 10 (no distress) to 50 (severe distress). Self-rated health and QoL were based on two questions, “In general, how would you rate your overall health/quality of life?”, followed by response options of ‘excellent’, ‘very good’, ‘good’, ‘fair’, and ‘poor’. In stratification by level of impact of pain, participants were considered to have: ‘high-impact pain’ if they answered ‘moderately’ or ‘extremely’; ‘low-impact pain’ if they answered ‘a little bit’ or ‘quite a bit’; and ‘no impact of pain’ if they answered ‘not at all’.

### Other variables

Sociodemographic and health characteristics were those reported on the baseline or Wave 2 questionnaire and included age, sex, education, region of residence, country of birth, body mass index (BMI), physical activity, smoking status, number of alcoholic drinks per week, self-reported comorbidities, medications, and the need for help with daily tasks (Table 1).

### Statistical methods

Descriptive statistics summarised demographic and clinical data. Modified Poisson regression models estimated age- and sex-adjusted prevalence ratios (PRs) and 95% confidence intervals (CIs) for bodily pain and high-impact pain in those with cancer versus those without, overall and for each cancer type. Models were adjusted for age and sex (where applicable); further statistical adjustments were not done as the objective of the study was to compare prevalence and lived experiences rather than establish causality. Analyses focused on high-impact pain. PRs were also estimated with further stratification for clinical characteristics of cancer (time since diagnosis, cancer stage, recent cancer treatment) and population subgroups; categories with at least 10 participants with the outcome of interest were analysed. Those with missing outcome data (*n* = 3,224, 2.6% for bodily pain; *n* = 3,225, 2.6% for high-impact pain) were excluded from the corresponding analyses.

To quantify the combined relation of cancer and level of impact of pain to severe physical functioning limitations (MOS-PF score < 60) [17], moderate/high psychological distress (K10 score 16–50) [18], fair/poor self-rated health, and fair/poor QoL, PRs for each of these adverse health outcomes were estimated among participants with and without a cancer diagnosis further stratified by the level of impact of pain. Those with no impact of pain and without cancer were used as the reference group.

Analyses were carried out using SAS, version 9.4. [19].

## Results

### Characteristics of the study population

Compared to 106,345 participants without cancer, the 16,053 cancer survivors were on average older, more likely to be male, more likely to have doctor-diagnosed cardiovascular disease, and were similar with respect to other characteristics (Table 1).

**Table 1:** Characteristics of the study population

	Cancer survivors (*n* = 16,053)	Participants without cancer (*n* = 106,345)	Total (*n* = 122,398)
**Age group**, % (n)			
45–64 years	27 (4399)	51 (53,927)	58,326
65–79 years	53 (8568)	39 (41,943)	50,511
≥ 80 years	19 (3086)	10 (10,475)	13,561
**Male**, % (n)	56 (8981)	43 (46,178)	55,159
**University degree**, % (n)	25 (4057)	29 (30,992)	35,049
**Residing in major cities**, % (n)	49 (7814)	48 (50,809)	58,623
**Australian born**, % (n)	80 (12,833)	78 (82,487)	95,320
**Body mass index, kg/m**^**2**^, % (n)			
Overweight (25 to < 30)	37 (5891)	35 (37,388)	43,279
Obese (30 to 50)	21 (3443)	22 (22,892)	26,335
**Highest physical activity tertile**, % (n)	32 (5117)	35 (37,416)	42,533
**Smoking status**, % (n)			
Current	3 (475)	4 (4329)	4804
Former	40 (6395)	35 (36,874)	43,269
** ≥ 15 alcoholic drinks per week**, % (n)	14 (2264)	13 (14,117)	16,381
**Self-reported comorbidities**, % (n)			
Cardiovascular disease	29 (4595)	21 (22,259)	26,854
Diabetes	12 (1980)	10 (10,178)	12,158
Parkinson's disease	1 (148)	1 (740)	888
Asthma	11 (1812)	12 (12,960)	14,772
Osteoarthritis	20 (3172)	18 (19,630)	22,802
Depression	14 (2215)	15 (16,075)	18,290
Anxiety	10 (1527)	11 (11,655)	13,182
**Medications**, % (n)			
Paracetamol without codeine	28 (4473)	26 (27,416)	31,889
Paracetamol with codeine	6 (970)	6 (6798)	7768
Aspirin for other reasons	5 (818)	4 (4608)	5426
**Need help with daily tasks**, % (n)	11 (1752)	7 (7312)	9064

Percentages are out of column totals which include missing values for region of residence (10%), body mass index (16%), medications (13%), and other variables (≤ 3%). There were no missing values for age or sex

Body mass index (kg/m^2^) was based on self-reported height and weight

Highest physical activity tertile was based on physical activity sessions per week, weighted for intensity

Comorbidities were based on responses to questions on “Has a doctor ever told you that you have…?”. Categories of self-reported comorbidities are not mutually exclusive and may overlap

Medications were based on responses to the question, “Have you taken any medications, vitamins or supplements for most of the last 4 weeks?”, followed by a list of medications with tick boxes. Regular pain-relieving medications included paracetamol without codeine, paracetamol with codeine, aspirin for heart disease and aspirin for other reasons. Categories of self-reported medications are not mutually exclusive and may overlap

### Clinical characteristics of cancer and cancer type

Prostate cancer (28.5%), breast cancer (19.4%), melanoma (15.7%), and colorectal cancer (11.5%), accounted for three-quarters of all cancers (Additional file 2: Table S2). Clinical characteristics varied by cancer type. The median time since diagnosis was 5.9 years, with 43.2% diagnosed in the five years prior to the survey. Participants with lung and oesophageal cancer were more likely to have been diagnosed within the previous year compared to those with other cancers. Localised disease was most common in survivors of melanoma and least common in colorectal cancer survivors. The majority of survivors had not received cancer treatment in the past month, except for those with multiple myeloma.

### Prevalence of bodily pain and high-impact pain

**Fig. 1:**
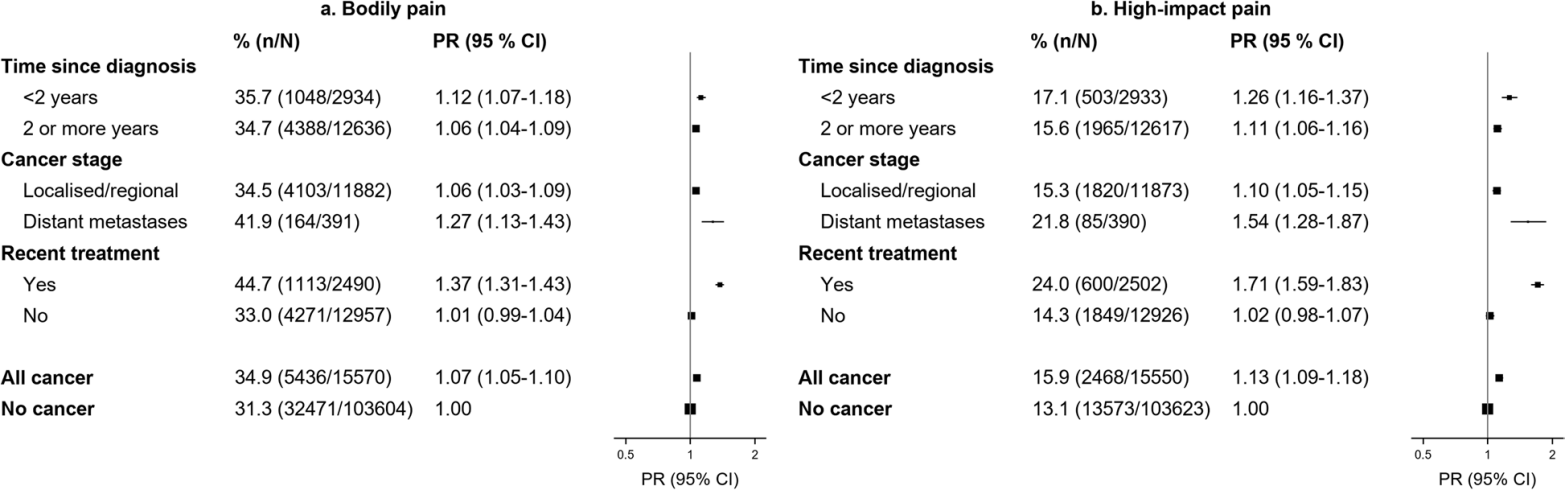
Prevalence of bodily pain and high-impact pain in cancer survivors versus individuals without cancer, according to clinical characteristics of cancer. CI: confidence interval; PR: prevalence ratio (adjusted for age and sex). Cancer staging is applicable to solid tumours only (ICD-10-AM diagnosis codes C00.0-C43.9 or C45.0-C80). Findings by cancer type are presented in Additional File 2: Tables S3-S5. Bodily pain: Participants were asked, “How much bodily pain have you had during the past 4 weeks?”, followed by response options of ‘none’, ‘very mild’, ‘mild’, ‘moderate’, ‘severe’, and ‘very severe’. Participants were considered to have bodily pain if they answered ‘moderate’, ‘severe’ or ‘very severe’. High-impact pain: Participants were asked, “During the past 4 weeks, how much did pain interfere with your normal work (including both work outside the home and housework)?”, followed by response options of ‘not at all’, ‘a little bit’, ‘quite a bit’, ‘moderately’, and ‘extremely’. Participants were considered to have high-impact pain if they answered ‘moderately’ or ‘extremely’

Overall, 34.9% (5,436/15,570) of cancer survivors reported bodily pain, compared to 31.3% (32,471/103,604) of participants without cancer (age- and sex-adjusted PR = 1.07; 95% CI = 1.05–1.10) (Fig. 1). Overall, 15.9% (2,468/15,550) of cancer survivors and 13.1% (13,573/103,623) of those without cancer had high-impact pain (1.13 [1.09–1.18]; Fig. 1).

### Variations by clinical characteristics

Compared to participants without cancer, the PRs for high-impact pain were 1.26 (1.16–1.37) for cancer survivors diagnosed < 2 years versus 1.11 (1.06–1.16) for ≥ 2 years previously (Fig. 1). Corresponding PRs were: 1.10 (1.05–1.15) for localised disease; 1.54 (1.28–1.87) for metastatic disease; 1.71 (1.59–1.83) for cancer treatment in the previous month; and 1.02 (0.98–1.07) for cancer survivors not receiving treatment in the previous month (Fig. 1). Findings were similar for bodily pain (Fig. 1).

### Variations by cancer type, overall

**Fig. 2:**
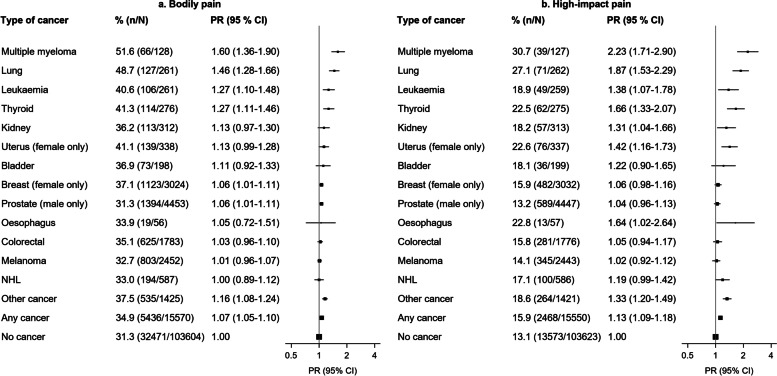
Prevalence of bodily pain and high-impact pain in cancer survivors versus individuals without cancer, for 13 cancer types. CI: confidence interval; NHL: non-Hodgkin’s lymphoma; PR: prevalence ratio (adjusted for age and sex). Cancer types were classified as breast (ICD-10-AM diagnosis code C50, women only), prostate (C61, men only), lung (C33–C34), melanoma (C43), colorectal (C18–C20), non-Hodgkin’s lymphoma (C82–C86), kidney (C64), oesophagus (C15), uterus (C54–C55, women only), bladder (C67), thyroid (C73), leukaemia (C91–C95), multiple myeloma (C90.0), and ‘other cancers’ (Additional File 2: Table S1). Interaction with sex was observed for kidney cancer; prevalence of moderate/severe/very severe bodily pain was 27.8% (PR 95% CI: 0.95 [0.76–1.19]) in men and 50.9% (PR 95% CI: 1.40 [1.16–1.68]) in women, and prevalence of high-impact pain 12.6% (PR 95% CI: 1.00 [0.69–1.45]) among men versus 28.1% (PR 95% CI: 1.78 [1.32–2.39]) among women

High-impact pain was greatest in those with multiple myeloma (PR = 2.23; 95% CI = 1.71–2.90) and lung cancer (1.87 [1.53–2.29]), followed by thyroid, oesophagus, uterus, leukaemia, and kidney cancer (Fig. 2). The risk of high-impact pain in participants with melanoma and breast, prostate, and colorectal cancer did not differ significantly from risk in participants without cancer. Findings were similar for bodily pain (Fig. 2).

### Variations by cancer type and clinical characteristics

Considering both clinical characteristics and cancer type, multiple myeloma and lung cancer survivors experienced the greatest high-impact pain in both categories of time since diagnosis (Additional file 2: Table S3). Treatment for cancer in the past month was associated with significantly greater high-impact pain for all cancer types able to be investigated; prevalences were at least two times for thyroid cancer, multiple myeloma, lung cancer, and uterus cancer (Additional file 2: Table S4). Lung cancer survivors experienced the greatest high-impact pain in both groups of cancer stage (Additional File 2: Table S5). Findings were similar for bodily pain (Additional file 2: Tables S3-S5).

### Variations by sociodemographic characteristics

**Fig. 3:**
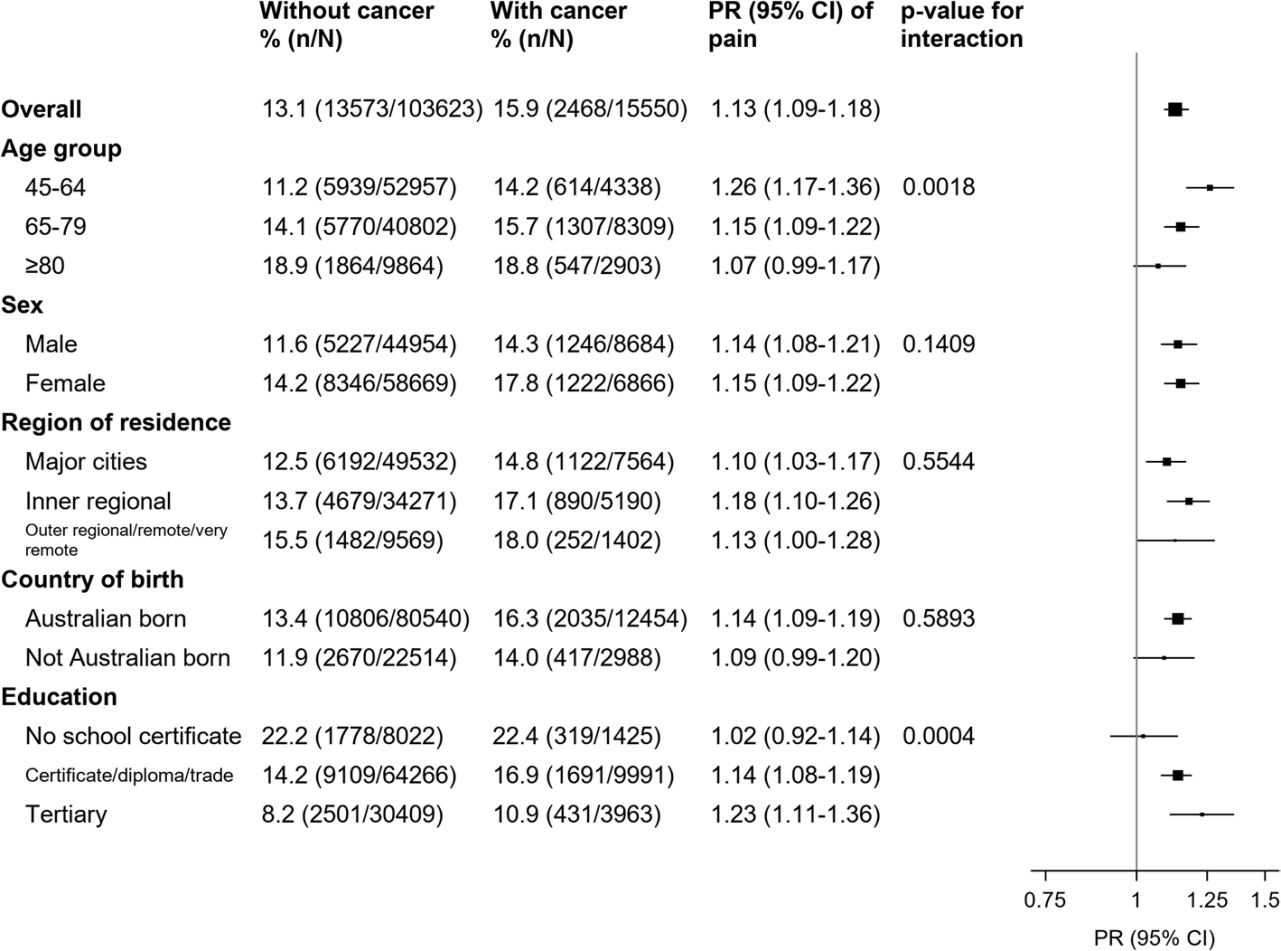
Prevalence of high-impact pain in cancer survivors versus individuals without cancer, in a range of population subgroups. CI: confidence interval; PR: prevalence ratio (adjusted for age and sex)

Greater levels of high-impact pain in those with versus without cancer were observed across most of the demographic groups examined (Fig. 3). Prevalence ratios were greater in younger versus older age-groups (Fig. 3; p_interaction_ = 0.0018) and in those with higher educational qualifications (p_interaction_ = 0.0004). No significant variation was observed according to sex, region of residence, or country of birth. Findings were similar for bodily pain (Additional file 2: Figure-S1).

### Joint relationship of cancer and pain to other adverse outcomes

**Fig. 4:**
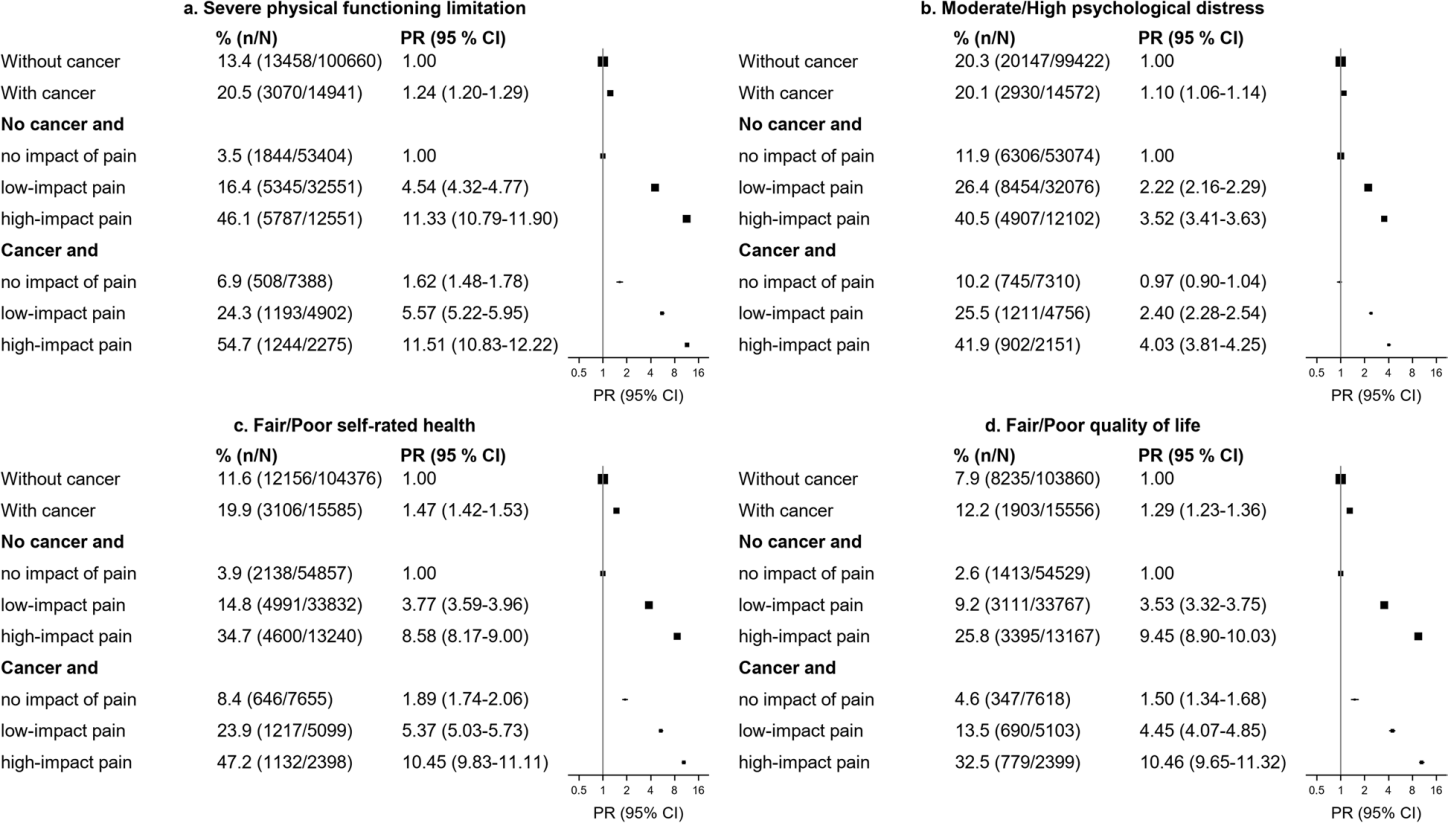
Age- and sex-adjusted prevalence ratios for person-centred outcomes in people with and without a history of cancer and according to level of impact of pain. CI: confidence interval; PR: prevalence ratio (adjusted for age and sex). Severe physical functioning limitation was defined as a Medical Outcomes Study Physical Functioning (MOS-PF) score < 60 [15, 17]. The score was elicited from self-reported data on limitations in the ability to perform moderate and vigorous physical activities and tasks such as: lifting or carrying shopping; climbing stairs; walking; bending, kneeling or stooping; and bathing or dressing. Scores can range from 0 to 100, where a lower score indicates more severe functioning limitation [17]. Psychological distress was assessed using the Kessler 10 (K10) scale [16]. Respondents indicated the frequency of symptoms experienced in the past four weeks, from 1 ‘none of the time’ to 5 ‘all of the time’. Scores can range from 10 (no distress) to 50 (severe distress). Moderate/high psychological distress was defined as a K10 score between 16 and 50 [18]. Level of impact of pain: Participants were asked, “During the past 4 weeks, how much did pain interfere with your normal work (including both work outside the home and housework)?”, followed by response options of ‘not at all’, ‘a little bit’, ‘quite a bit’, ‘moderately’, and ‘extremely’. They were considered to have: ‘high-impact pain’ if they answered ‘moderately’ or ‘extremely’; ‘low-impact pain’ if they answered ‘a little bit’ or ‘quite a bit’; and ‘no impact of pain’ if they answered ‘not at all’

In participants with and without cancer, the prevalence of severe physical functioning limitations, moderate/high psychological distress, fair/poor self-rated health, and fair/poor QoL increased substantially with increasing levels of impact of pain (Fig. 4). For example, around 45–55% of those with high-impact pain had severe physical functioning limitations and 40% were experiencing moderate/high psychological distress. Among participants without high-impact pain, cancer survivors had somewhat elevated PRs compared to participants without cancer for severe physical functioning limitations, and fair/poor self-rated health and QoL (50–89% higher), but no significant difference was observed for moderate/high psychological distress.

## Discussion

This study finds that pain is common among Australian community-dwelling adults, with around one-third of participants reporting moderate to very severe bodily pain and around one-in-eight reporting moderate or extreme high-impact pain. Cancer survivors have somewhat elevated levels of pain in general, with pain varying markedly by cancer type. Greater levels of pain were observed in survivors of blood cancers such as multiple myeloma and leukaemia and poor prognosis tumours such as lung cancer, and lesser or no elevations in pain were observed for many common cancers such as those of the breast, prostate, and colorectum, as well as melanoma.

Pain was greater in those with a more recent diagnosis of cancer, those with advanced disease, and those reporting recent cancer treatment, compared to other survivors and to participants without cancer. Cancer survivors not reporting recent cancer treatment, who constitute the majority of survivors, had levels of high-impact pain similar to the general population. This study, to our knowledge, is the first to consider different cancer types and stages separately, to compare people with and without cancer, and to emphasise pain that has an impact on work and daily life.

Cancer is a highly heterogeneous condition with treatment options depending on factors such as type, location, and stage; cancer pain during and after the treatment phase could be secondary to cancer, resulting from cancer treatment, or for other reasons [4]. We identified 17 previous studies which included comparisons with the general population or a control group, but differences in methods made direct comparisons difficult (Additional file 1). Only four studies were similar large-scale studies using comparable outcome measures; these did not include analyses by clinical characteristics, but indicated higher prevalence of bodily pain [20–22] and high-impact pain [23] in cancer survivors compared to people without cancer. Previous findings of higher odds of chronic pain in cancer survivors than those without a cancer history, [20] including in survivors of kidney, lung, colorectal, uterus cancers and haematologic cancers, as well as comparable odds in survivors of bladder, prostate, breast cancers and melanoma, are consistent with results of this study; higher odds of chronic pain associated with cancer of the cervix, head and neck, ovary and sarcoma are to some extent reflected in the observed elevated levels of outcomes in the 'other cancer' group in this study.

Pain burden at the population-level peaks in late middle age, attributable to injury and the presence of medical and musculoskeletal conditions such as arthritis [24]. For cancer survivors, cancer type and treatments received are likely to contribute additionally to pain. This includes cancer-related bone and other tissue pain arising from the underlying disease, as well as pain arising from treatments such as chronic post-surgical pain, chemotherapy-induced peripheral neuropathy, and radiation-induced pain [25, 26]. Findings regarding the association between pain and recent treatment for cancer are consistent with previous research (Additional file 1). Invasive treatments for lung cancer [27] are likely to contribute to the high prevalence of pain and comorbidity in this tumour type. A range of factors are likely to contribute to the higher prevalence of bodily pain and high-impact pain among survivors of multiple myeloma. These include osteolytic bone lesions – with bone pain affecting up to 90% of survivors – chemotherapy-induced peripheral neuropathy, and neuralgia caused by reactivation of herpes zoster virus [28]. Multiple myeloma survival has increased considerably over the last few decades and active disease episodes are interrupted by longer periods with disease inactivity, when many patients continue to live with intense pain interfering with their daily activities [28]. Specific cancer types, including multiple myeloma, lung, prostate, breast, and kidney cancer, are also associated with painful bone metastases [28]. The exact reasons for elevated pain in survivors of thyroid cancer are unclear [29, 30]. These findings reinforce the need for pain assessment and targeted pain management strategies, tailored to cancer type and stage of cancer progression or recovery.

While these results demonstrate elevated levels of pain and high-impact pain among cancer survivors, they also indicate that for people with many common cancer types – including breast, prostate, and colorectal cancers and melanoma – levels of pain do not differ, on average, from those of the rest of the population. Similarly, cancer survivors not receiving treatment within the past month – who constitute the majority of survivors – do not have elevated pain levels. Findings regarding greater differentials in high-impact pain in cancer survivors versus people with no history of cancer at younger ages could be due to lower levels of background pain in the general population at younger ages, higher multimorbidity in older ages, as well as greater acceptance of cancer pain among older survivors and better coping strategies [31].

Overall, the findings from this study and the broader evidence [13] highlight the need for continuous assessment of physical and psychological morbidity, and of the well-being of cancer survivors, better management of pain, and targeted intervention to enhance long-term function and high QoL. Current pain management is often fragmented and not integrated appropriately into routine care. Our findings highlight a need to ensure that interventions to address pain are available to people during and after cancer treatment. The findings suggest that people diagnosed with cancer, particularly those with blood cancers and lung cancer, may benefit from monitoring during and after completion of treatment, for cancer-related pain. Cultural factors have been shown to influence experiences of pain and this is being explored in a future paper within the 45 and Up Study. Overall, 80% of participants in this study were born in Australia and around 90% report speaking only English in the home. Consideration of cultural factors is important in understanding and managing pain in people with and without cancer.

Our findings indicate that physical functioning limitations, psychological distress, and fair/poor self-rated health and QoL are three to 11- fold in those with versus without high-impact pain, both in people with and without cancer. These general person-centred outcomes varied to a greater extent according to level of high-impact pain than according to whether or not participants had cancer. Based on evidence from this study as well as previous research, pain and its interference with daily life are likely key drivers of physical functioning limitations, psychological distress, and reduced QoL, in people with and without cancer [32, 33]. This may be influenced by the bidirectional and mutually reinforcing relationship between pain and distress. Further research into the intersection between physical pain and mental health is urgently needed, particularly for those who experience the greatest burden from pain.

This study is large-scale and population-based, with linkage to cancer registry data. This enabled comprehensive comparative quantification of pain experienced by individuals living with cancer in the community in relation to background experiences of pain in the broader community, including in different population subgroups with varying cancer characteristics. Participants in cohort studies, including this study, are healthier than the general population [34]. Hence, while our absolute estimates of cancer prevalence and pain may not be directly representative, PRs, which are based on internal comparisons, are likely to be generalisable [35]. Temporal information on pain in relation to incident cancer diagnosis was not available as information on pain was collected only in the follow-up survey. Pain is based on self-report; linked clinical or qualitative interview data were not available to further understand how participants’ pain was addressed and whether they were currently able to access help from cancer or primary care providers. The survey period (2012–2015) meant that participants were unlikely to have received emerging therapies such as immunotherapy.

## Conclusions

Our results highlight that pain is common in the community and more common among cancer survivors than those without cancer. Pain prevalence varies by cancer type; survivors of multiple myeloma, leukaemia, and lung cancer had the highest prevalence of bodily pain and high-impact pain. No elevation in pain was observed for some cancer types, including breast, prostate, colorectum, bladder, and melanoma. People with pain experience an increased risk of poor physical functioning, mental health, and QoL, regardless of whether or not they have had cancer. Targeted interventions to mitigate and prevent pain, including high-impact pain, are needed to improve well-being after cancer.

### Supplementary Information

**Additional file 1.**  Brief literature review of cancer and pain/pain interference.

**Additional file 2:** **Table S1.** ICD-10-AM codes for cancers included in the ‘other cancer’ group. **Table S2.** Clinical characteristics of cancer by cancer type. **Table S3.** Prevalence of bodily pain and high-impact pain by cancer type and time since cancer diagnosis. **Table S4. **Prevalence of bodily pain and high-impact pain by cancer type and recent treatment for cancer. **Table S5. **Prevalence of bodily pain and high-impact pain by cancer type and stage. **Figure S1.** Prevalence of bodily pain in cancer survivors versus individuals without cancer, in a range of population subgroups.

## Data Availability

This research was completed using data collected through the 45 and Up Study (www.saxinstitute.org.au). The 45 and Up Study is managed by the Sax Institute in collaboration with major partner Cancer Council NSW; and partners: the National Heart Foundation of Australia; NSW Ministry of Health; and Australian Red Cross Lifeblood. Data supporting the findings from this study are available from the Sax Institute, the NSW Department of Health, and the Australian Bureau of Statistics, with data linkage conducted by the NSW Centre for Health Record Linkage. Restrictions apply to the availability of these data, which were used under license for the current study, and so are not publicly available. Data are available from the authors upon reasonable request and with permission of the Sax Institute (www.saxinstitute.org.au) and the NSW Department of Health.

## Abbreviations

BMI: Body mass index
CCR: Central Cancer Registry
CI: Confidence interval
ICD: International Classification of Diseases
K10: Kessler 10
PR: Prevalence ratio
QoL: Quality of life
MOS-PF: Medical Outcomes Study Physical Functioning
NHL: Non-Hodgkin’s lymphoma
NSW: New South Wales
