# Supplementary material for: Pain and its interference with daily living in relation to cancer: a comparative population-based study of 16,053 cancer survivors and 106,345 people without cancer
Source: BMC Cancer. 2023 Sep 13;23:774. doi: 10.1186/s12885-023-11214-5 (PMC10498633; doi:10.1186/s12885-023-11214-5)
Supplement: Supplementary file 1 — Additional file 1. Brief literature review of cancer and pain/pain interference. [file 12885_2023_11214_MOESM1_ESM.pdf]

Additional File 1: Brief literature review of cancer and pain/pain interference

Table of Contents

Aim 2
Methods 2
Inclusion criteria 2
Exclusion criteria 3
Database search 4
Search strategy 4
Screening 5
Data extraction 5
Results 7
Key findings 7
Characteristics of the studies 8
Pain – any cancer (unspecified) 9
Pain – any cancer (multiple diagnoses, grouped or single diagnoses compared) 10
Pain – individual cancer type 17
Breast 17
Colorectal 18
Prostate 18
Thyroid 19
Leukaemia 19
Lung 19
Melanoma 20
Multiple myeloma 20
Non-Hodgkin's lymphoma 20
Kidney, oesophagus, uterus, bladder cancer 20
Evidence on pain in relation to demographic and clinical characteristics in cancer survivors (age, time since diagnosis, cancer stage, treatment type and time since treatment) 21
Pain interference 28
Discussion 30
Research gaps 31
References 34

Aim

The primary aim of this systematic literature review was to review and summarise the worldwide published evidence on the prevalence of pain and pain interference (also referred to as 'high-impact' pain) in adult cancer survivors, compared to those without cancer, for a range of common cancer types. The secondary aim was to explore the relationship between demographic and clinical factors and pain/pain interference in cancer survivors.

Methods

The database search was based on the original data analysis plan (DAP), which included the primary outcomes of pain and pain interference, along with additional outcomes of psychological distress, quality of life, disability and fatigue. After several team meetings, the DAP was refined, and the focus of the review was restricted to pain-related outcomes only. Studies reporting on survivors with single, multiple, and unspecified cancer types were included. Whilst the aim of the review was to determine relative estimates of pain and pain interference (cancer survivors compared to those without cancer), studies without a comparison group were included and had basic information extracted (title, author(s), year of publication, country, sample size, study population, study design), as opposed to detailed data extraction. Evidence on variations in pain/pain interference according to demographic factors (age and sex) and clinical characteristics (cancer type, stage of cancer, time since diagnosis, treatment: type; duration; and time since) in cancer survivors were also reviewed.

Inclusion criteria

Study design: Published, peer reviewed, original observational studies (cross-sectional, cohort, case-control).

Population: Individuals diagnosed with cancer as adults (≥18 years of age). Limited to prevalent cancers in Australia: breast, prostate, lung, melanoma, colorectal, non-Hodgkin's lymphoma, kidney, oesophagus, uterus, bladder, thyroid, leukaemia, and multiple myeloma.

Comparison: Individuals without a cancer diagnosis.

Outcome: Prevalence of pain and/or pain interference. Evidence on variations in pain and/or pain interference according to clinical and demographic characteristics.

Timing: All years

Setting: Any country

Language: Articles published in English

Exclusion criteria

Study design: Grey literature, conference abstracts, letters, editorials, correspondence, opinion pieces, government reports, position statements, systematic reviews, qualitative research, intervention studies (e.g. randomised controlled trials), and protocols.

Population: Individuals without a cancer diagnosis, adolescent/childhood cancer survivors (<18 years of age at diagnosis), individuals diagnosed with a form of cancer not listed in the inclusion criteria (e.g. testicular, head and neck, sarcoma, endometrial, ovarian, cervix), and caregivers of cancer survivors.

Outcome: Prevalence of person-centred or other outcome unrelated to pain and/or pain interference (e.g. insomnia, employment, health care and

medication utilisation, unmet supportive care needs, quality of life, psychological distress [anxiety, depression, post-traumatic stress], fatigue, disability, symptom burden, cognitive impairment, fear of cancer recurrence, level of functioning, sexual health and satisfaction, spiritual/emotional health, and symptom cluster studies [pain grouped with other outcomes]).

Timing: No exclusion criteria

Setting: No exclusion criteria

Language: Articles not published in English

Other: Duplicated data, unavailable full text

Database search

PubMed, Scopus, and Web of Science were searched on 23 March 2021 to identify relevant literature published up until that date. An additional search, using the same databases and search strategy, was conducted on 28 February 2022 to retrieve articles published since the first literature search.

Search strategy

	PubMed	Scopus	Web of Science
**Population terms**	1. Cancer survivors [MeSH Terms]	1. "Cancer survivor" [TAK]<br>2. Survivor [TAK]	1. "Cancer survivor" [TS]<br>2. Survivor [TS]
**Cancer terms**	1. Neoplasms [MeSH Terms]<br>2. Cancer [Title/Abstract]	1. Neoplasm [TAK]<br>2. Cancer [TAK]	1. Neoplasm [TS]<br>2. Cancer [TS]
**Outcome terms**	1. Pain [MeSH Terms]<br>2. Pain [Title/Abstract]<br>3. Psychological distress [MeSH Terms]<br>4. Fatigue [MeSH Terms]<br>5. Fatigue [Title/Abstract]<br>6. Health status [MeSH Terms]<br>7. Disabled persons [MeSH Terms]	1. Pain [TAK]<br>2. "Psychological distress" [TAK]<br>3. Fatigue [TAK]<br>4. "Health status" [TAK]<br>5. "Quality of life" [TAK]<br>6. "Functional status" [TAK]<br>7. Disab* [TAK]	1. Pain [TS]<br>2. "Psychological distress" [TS]<br>3. Fatigue [TS]<br>4. "Health status" [TS]<br>5. "Quality of life" [TS]<br>6. "Functional status" [TS]<br>7. Disab* [TS]
**Other terms**	1. Prevalence [MeSH Terms]	1. Prevalence [TAK]	1. Prevalence [TS]

	2. Prevalence [Title/Abstract]		
**Filters/indexes**	1. English	1. English	1. English<br>2. SCI-EXPANDED<br>3. ESCI

MeSH: Medical Subject Headings; **TAK**: Title-Abstract-Key; **TS**: Topic (Title, Abstract, Author Keywords, Keywords Plus®)

Screening

Results from the search were imported into EndNote version 20. Duplicates were removed in EndNote and the remaining articles were imported into the web-based platform Covidence. Duplicates that were missed by EndNote were subsequently removed by Covidence. The titles and abstracts of the articles were independently screened by two reviewers (SB and ST). Similarly, both reviewers independently screened the full texts. At both stages, conflicts were resolved through discussion between the two reviewers. Articles that met all of the inclusion criteria at full text screening were included in the review. *Figure 1* highlights the number of articles at each stage of the study selection process.

Data extraction

Data were extracted from the publications into a pre-specified data extraction template in Microsoft Excel. One reviewer extracted data from half of the identified studies, and the other reviewer extracted data from the other half. Each reviewer checked the others' work for accuracy and missing data.

Figure 1. Study flow diagram

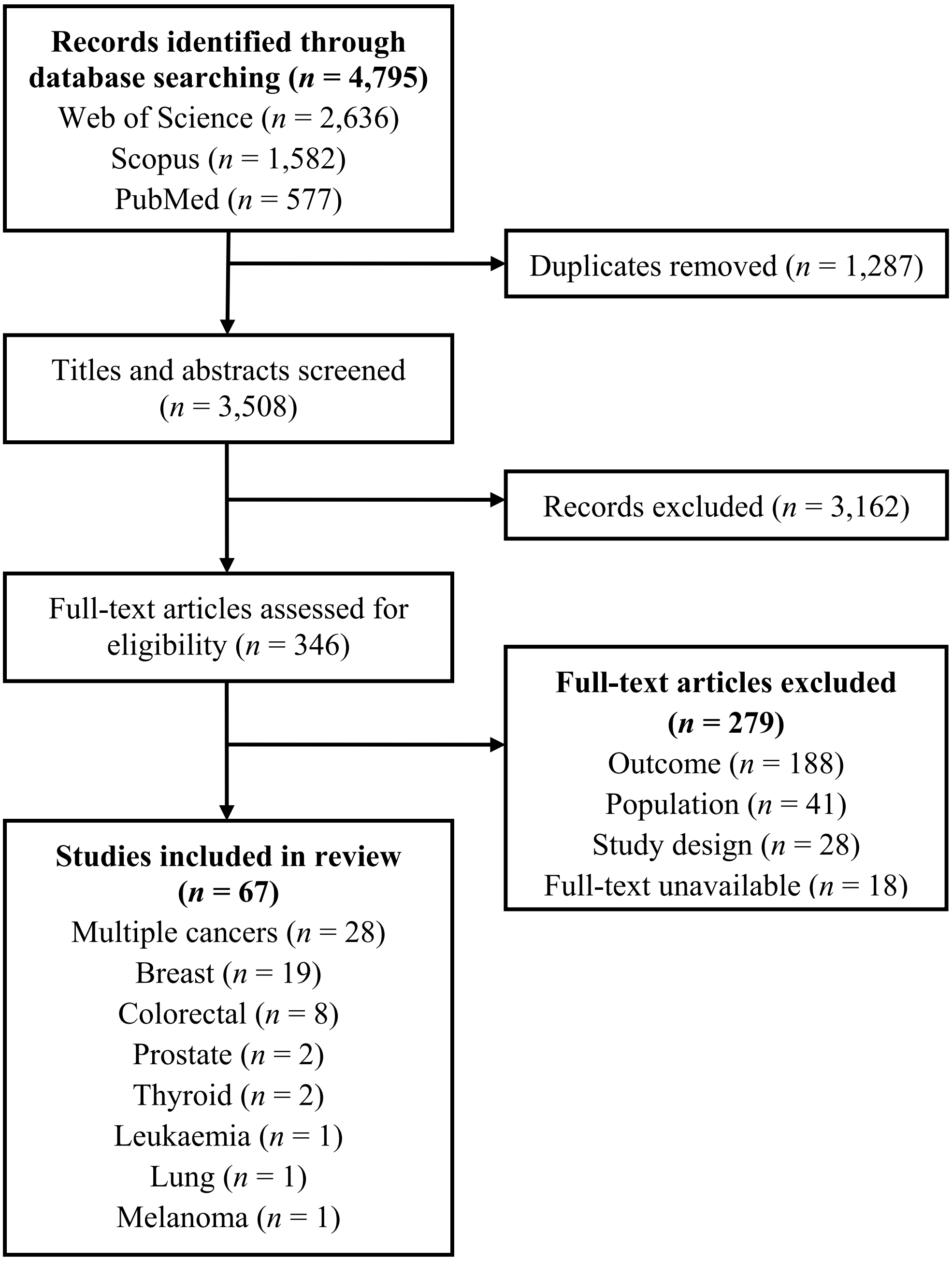

Results

Key findings

1. A total of 67 articles were included in the review; most involved small sample sizes and/or focused on single common cancer types such as breast, prostate, and colorectal cancer. Very few studies were identified in survivors of kidney, oesophagus, leukaemia, and multiple myeloma cancers. Over 50% were published in 2017 or earlier. Given that recruitment/assessment often occurs many years before studies are published, there is a lack of contemporary data in the context of rapid advances in treatment for cancer and pain management.
2. Studies varied in the way pain was measured in terms of severity/impact (ranging from dichotomous general pain [1] to pain-interference on a five-point scale [2]), recall period (from one week [2] to two years [3]), and type of pain (e.g. low back pain, [4, 5] head/neck pain [5]).
3. Several studies investigated pain outcomes in cancer survivors who had received specific treatments/medications (e.g. thyroidectomy, mastectomy) which limits ability to be directly compared with our analysis. Follow-up time varied, with some studies focusing on post-surgery pain and some conducting follow-up several years later.
4. In several studies, pain was only assessed at certain body parts or regions, e.g. breast pain several months following breast conserving surgery, and head/neck and low back pain. In one study, pain was grouped with cramping and abdominal discomfort.
5. Only 17 studies (16 investigating pain [1, 2, 5-18] and one investigating pain interference [3]) included a control/comparison group without cancer. The number of cancer survivors ranged from 121 to 7,565. Even in the relatively larger studies with 1,000 or more cancer survivors, differences in methods made direct comparisons difficult; for

example, some studies [2, 6] reported mean pain scores, one study [5] investigated head/neck and low back pain, and another used breast cancer survivors as the reference group for analysis pertaining to time since diagnosis [7]. Similar large-scale studies indicated higher prevalence of pain [1, 8, 10] and pain interference [3] in cancer survivors relative to controls.

6. There were only two studies published in Australia, and neither included a control/comparison group [19, 20].
7. Only four previous studies included comparison with the general population on comparable outcome measures; these did not include analyses by cancer type or clinical characteristics, but indicated higher prevalence of bodily pain (1,8,10) and high-impact pain (3) in cancer survivors compared to people without cancer.

Characteristics of the studies

Twenty-eight studies involved multiple cancer types [2, 3, 5-10, 19, 21-39], where either survivors of different single cancer diagnoses were compared; survivors of multiple cancer diagnoses were compared with survivors of single cancer diagnoses and no cancer; or several cancers were grouped as one 'cancer' group. Nineteen studies were in breast cancer survivors [4, 13, 14, 40-55], eight were in colorectal cancer survivors [20, 56-62], two each were in prostate [15, 63], and thyroid [16, 64] cancer survivors, one each were in leukaemia [17], lung [65], melanoma [18], multiple myeloma [66], and non-Hodgkin's lymphoma survivors [67], and three were in survivors of unspecified cancer types [1, 11, 12].

Forty-seven were cross-sectional studies [1-13, 16, 18, 19, 21-24, 28, 31, 33-36, 39-41, 43-45, 47, 50, 51, 54-57, 59-66], 17 were cohort studies [14, 20, 25, 27, 29, 30, 32, 37, 38, 42, 46, 48, 49, 52, 53, 58, 67], two were case-control studies [15, 17], and in one study the design was unclear [26].

Seventeen studies (16 investigating pain [1, 2, 5-18] and one investigating pain interference [3]) included a cancer-free control/comparison group, while 50 did not.

Twenty-six studies were from the United States (US) [1, 3, 5, 7, 8, 10, 11, 13, 18, 24, 25, 27, 28, 30, 32, 33, 35, 40-42, 52, 54, 58, 59, 62, 66], four each were from Germany [6, 16, 34, 64] and the Netherlands [2, 15, 60, 61], three each were from Denmark [29, 48, 50], Norway [4, 22, 36] and South Korea [9, 31, 67], two each were from Australia [19, 20], Brazil [45, 55], Canada [14, 49], Ireland [56, 63], Italy [17, 46], Spain [43, 53], and one each was from China [57], France [21], Iceland [39], Israel [47], Malaysia [37], New Zealand [44], Pakistan [51], Portugal [26], Taiwan [65], Turkey [23], Israel/European region (seven nations) [12], and the Asian region (eight nations) [38].

Four studies (three cross-sectional [3, 19, 22] and one cohort [58]) were identified that assessed pain interference.

Pain – any cancer (unspecified)

Three cross-sectional studies were identified which did not specify the cancer types of the included population [1, 11, 12].

In the first study, 403 cancer survivors aged 20 to 64 years from the 2000-2002 Centers for Disease Control and Prevention (CDC) Behavioral Risk Factor Surveillance System (BRFSS) were compared with 224,606 individuals with no limitations (limitations were defined as any major physical health, mental or emotional problem/impairment, e.g. cancer, depression, anxiety, cardiovascular disease) [11]. The mean number of 'painful days' in the past 30 days was 13.1 (95% CI: 10.8, 15.4) for cancer survivors and 1.2 (95% CI: 1.1, 1.3) for those with no limitations [11].

The second study used data from the Health and Retirement Study (HRS) in the US, and assessed the prevalence and odds ratio (OR) of pain in 2,161 community-dwelling adult cancer survivors (≥51 years of age) compared with 15,049 community-dwelling adults without cancer [1]. There was a statistically significant difference ($p<0.0001$) in response to the question 'are you often troubled with pain?', with 33% of cancer survivors and 29% of the control group,

responding affirmatively. The OR for pain in cancer survivors relative to those without cancer was 1.15 (95% CI: 1.03, 1.28; p<0.01), adjusting for age, sex, race, educational level, insurance status, and coexisting medical conditions [1].

The third cross-sectional study was a multi-centre study across seven European nations and Israel, using data from the Services and Health for Elderly in Long TERm Care (SHELTER) study [12]. The study was conducted between 2009 and 2011 in nursing home residents who had a mean (SD) age of 83.4 (9.4) years. There were 442 cancer survivors and 3,698 individuals without cancer, and the primary cancer tumour was only known in 56 participants (n=11 breast cancer was the most prevalent of those known). Pain was defined as the visible presence of pain signs (defence reactions if touched, teeth grinding, grimacing, other non-verbal cues). There was a statistically significant difference in pain prevalence between the groups, with 46.8% and 34.8% of cancer survivors and control, respectively, displaying pain (p<0.001) [12].

Pain – any cancer (multiple diagnoses, grouped or single diagnoses compared)

Twenty-eight studies involving multiple cancer types – grouping multiple cancers together under one 'cancer' banner, comparing single cancer diagnoses or comparing survivors of multiple, single and no cancer diagnoses – were included in the review [2, 3, 5-10, 19, 21-39]. Twenty were cross-sectional [2, 3, 5-10, 19, 21-24, 28, 31, 33-36, 39], seven were cohort [25, 27, 29, 30, 32, 37, 38] and one was unclear [26]. Only studies including meaningful comparisons are described further (many studies which grouped several cancers did not have a control group and are therefore not presented in the narrative synthesis).

The first study assessed chronic neuropathic pain (CNP) in a group of 969 survivors of several cancer types aged 18-54 years at diagnosis (breast 57.5% of the sample, lung/aero-digestive tract 7.1%, rectum/colon 6.2%, bladder/kidney/prostate 4.9%, thyroid 10.3%, non-Hodgkin's lymphoma 3.4%, melanoma 6.8%, uterus/cervix 3.8%) in a French national cross-sectional

survey VIe après le CANcer (VICAN) [21]. CNP was assessed using the Douleur Neuropathique 4 (DN4) questionnaire at five-years post-diagnosis. The prevalence of CNP differed by sex and cancer type: breast (34%); lung and aero-digestive tract (women: 27%, men: 34%); colon-rectum (women: 22%, men: 19%); thyroid (women: 27%, men: 15%); lymphoma (women: 22%, men: 18%); melanoma (women: 22%, men: 25%); bladder and kidney (women: 32%, men: 24%); and uterus (29%). None of the comparisons between men and women were statistically significant. The prevalence of CNP did not differ between age groups (18-39, 40-49, 50-54; p=0.786), however the prevalence by treatment received (received chemotherapy: 36.3%, did not receive chemotherapy: 22.3%; p<0.001; received radiotherapy: 32.8%, did not receive radiotherapy: 24.8%; p=0.010) did differ significantly [21].

In Andrykowski et al., cross-sectional data from the 2009 US National Health Interview Survey (NHIS) were included, comparing 154 survivors of multiple cancers, 1,427 survivors of a single cancer and 25,004 individuals without a history of cancer (control), ≥18 years of age at diagnosis and ≥1 year since diagnosis, for cancer survivors [5]. Cancers included were bladder, breast, cervix, colon, kidney, lung, lymphoma, melanoma, ovary, prostate, skin (non-melanoma), skin (kind unknown), thyroid, uterus, and other. Pain was defined as the presence of head/neck or low back pain (yes or no) lasting one or more days during the past three months. The prevalence of head/neck pain was 40.9% for the multiple cancer group, 30.5% for the single cancer group and 26.0% for the control group. Compared to the control group, the multiple cancer group were 1.45 times as likely to experience head/neck pain (OR: 1.45; 95% CI: 1.23, 1.71; p<0.001). Compared to the single cancer group, the multiple cancer group were 1.65 times as likely to experience head/neck pain (OR: 1.65; 95% CI: 1.15, 2.36; p=0.006). The prevalence of low back pain was 38.3% for the multiple cancer group, 37.3% for the single cancer group and 27.9% for the control group. Compared to the control group, the multiple cancer group were 1.13 times as likely to experience low back pain (OR: 1.13; 95% CI: 0.95,

1.33; p=0.161). Compared to the single cancer group, the multiple cancer group were 1.02 times as likely to experience low back pain (OR: 1.02; 95% CI: 0.72, 1.45; p=0.898) [5].

In the US cross-sectional study by Gallaway et al., data was used from 2012, 2014, and 2016 Behavioral Risk Factor Surveillance System Cancer Survivorship Optional Module, to compare the weighted prevalence of pain among 12,019 survivors of different cancer types ≥18 years of age [24]. In response to the question 'do you currently have physical pain caused by your cancer or cancer treatment?', 28.3% (95% CI: 19.6, 38.9) of lung, 19.1% (95% CI: 16.3, 22.4) of female breast, 18.0% (95% CI: 12.6, 25.1) of leukaemia/lymphoma, 15.7% (95% CI: 10.0, 23.7) of colorectal, 5.3% (95% CI: 3.3, 8.3) of prostate, 2.7% (95% CI: 1.8, 4.1) of melanoma and 14.0% (95% CI: 11.5, 16.9) of 'all other' cancer survivors responded affirmatively, reported by weighted prevalence. The weighted prevalence of pain was greatest at younger ages (at diagnosis); 15.3% (95% CI: 12.2, 19.0) for 18-39-year-olds, 14.2% (95% CI: 11.8, 17.1) for 40-49-year-olds, 10.1% (95% CI: 8.7, 11.7) for 50-64-year-olds and 5.9% (95% CI: 4.6, 7.5) for ≥65-year-olds. The weighted prevalence of pain was greatest for groups closer to diagnosis, ranging from 13.0% (95% CI: 10.9, 15.4) for ≤5 years, 11.9% (95% CI: 9.7, 14.6) for 6-10 years, to 9.5% (95% CI: 8.0, 11.2) for >10 years since diagnosis [24].

In the Portuguese cohort study by Gonçalves et al., 85 survivors (≥30 years of age) of head and neck (n=13), digestive (n=43), genitourinary (n=5), breast (n=12), bone (n=2), hidden primary (n=1), central nervous system (CNS) (n=3) and synchronous (n=6) cancer were assessed [26]. Breast cancer was the only cancer relevant to this review, with six (50%) individuals reporting pain and six (50%) individuals reporting no pain [26].

Götze et al., a cross-sectional study published in Germany, measured pain in a population of 1,002 cancer survivors aged 18-85, five or ten years post-diagnosis [6]. The population was compared with European reference values for the pain questionnaire from six (n=16,151) European general population normative studies, matched on age and sex. The Quality of Life

Core Questionnaire of the European Organisation for Research and Treatment of Cancer (EORTC QLQ-C30) was used to assess pain on a 0-100 scale, with a higher score indicating higher pain. Cancer survivors were combined into one group and included prostate (25.5% of sample), breast (21.8%), gynaecological (9.5%), head and neck (7.8%), haematological (7.5%), skin (5.8%), kidney (5.0%), colon (4.7%) and other (12.5%). There was a statistically significant difference ($p<0.001$) between the cancer survivor population and the control population with respect to pain, with mean (SD) scores of 31.1 (32.6) and 21.1 (24.2) respectively. There was no difference between the five-year post-diagnosis group and the 10-year post-diagnosis group (five-year: 31.8 (32.8); 10-year: 29.8 (32.4); $p=0.369$) [6].

In the US cross-sectional study by Green et al., 194 adult (18-90 years of age) survivors of breast (n=84), colorectal (n=17), lung (n=9), prostate (n=81) or multiple myeloma (n=3) cancer were compared on pain outcomes [28]. Initial diagnosis was at least two years prior. The prevalence of current pain, current pain severity (assessed using the Brief Pain Inventory (BPI)-higher score equates to more severe pain), prevalence of pain since cancer and past pain severity (assessed using the BPI) were assessed. Percentage with current pain was 33.3% for multiple myeloma, 25.0% for breast cancer, 23.5% for colorectal cancer, 22.2% for lung cancer and 14.8% for prostate cancer. Current pain severity, mean (SD), was 4.00 ± 1.77 for lung cancer, 2.47 ± 1.71 for breast cancer, 2.06 ± 1.46 for colorectal cancer, 1.34 ± 1.07 for prostate cancer and 0.00 for multiple myeloma (n=1, no SD). Percentage with pain since cancer was 100.0% for multiple myeloma, 58.3% for breast cancer, 55.6% for lung cancer, 41.2% for colorectal cancer and 28.4% for prostate cancer. Past pain severity, mean (SD), was 7.44 ± 2.14 for multiple myeloma, 4.57 ± 1.95 for prostate cancer, 4.00 ± 2.10 for colorectal cancer, 3.49 ± 1.93 for breast cancer and 2.92 ± 2.04 for lung cancer. The authors stated that multiple myeloma had significantly higher ($p<0.01$) past pain severity than the other cancer types. The

proportion with pain since a cancer diagnosis was greatest for Stage IV (60.0%), Stage II (57.1%), Stage I (53.1%) and Stage III (50.0%) [28].

Mao et al. compared pain outcomes in 1,904 cancer survivors and 29,092 cancer-free individuals in their US cross-sectional study [7]. Data were from the 2002 NHIS and included ≥18-year-old breast (17.1% of sample), prostate (10.2%), colorectal (7.6%), cervix (9.3%), uterus (5.6%), melanoma (5.6%), multiple (9.5%) and other cancer survivors. Participants responded yes/no to the question 'during the past 12 months have you had recurring pain?' The unadjusted odds ratio for pain in cancer survivors compared to control was 2.44 (95% CI: 2.16, 2.74; $p<0.001$). The adjusted odds ratio for pain, when compared to a breast cancer reference group, was 1.82 for survivors of multiple cancers (95% CI: 1.21, 2.74; $p<0.05$), 1.22 for uterus cancer (95% CI: 0.75, 1.99), 1.10 for cervix cancer (95% CI: 0.71, 1.71), 1.06 for prostate cancer (95% CI: 0.68, 1.63), 0.95 for colorectal cancer (95% CI: 0.59, 1.53), 0.87 for melanoma (95% CI: 0.52, 1.46) and 1.13 for survivors of other cancers (95% CI: 0.83, 1.55). Compared to 0-1 year since diagnosis, the adjusted odds of pain for survivors 2-5 years since diagnosis was 0.83 (95% CI: 0.61, 1.13), 0.99 (95% CI: 0.70, 1.40) for 6-10 years and 1.06 (95% CI: 0.78, 1.45) for 11 years or greater. The models were adjusted for age, education, race/ethnicity and income (sex was not included as several cancer types are sex specific) [7].

In the Dutch cross-sectional analysis of a cohort study by Oertelt-Prigione et al., cancer survivors (n=5,339) from the Patient-Reported Outcomes Following Initial Treatment and Long-Term Evaluation of Survivorship (PROFILES) registry were compared with an age- and sex-matched cohort representative of the Dutch speaking population (n=389) [2]. The mean (SD) age at questionnaire was 66.8 (12) for male cancer survivors and 66.1 (13) for female cancer survivors. Colorectal (n=2,593), haematologic (n=1,751), basal/squamous cell (n=691) and thyroid (n=304) cancer survivors were combined into one group. The EORTC QLQ-C30 was used to assess pain on a 0-100 scale, with a higher score indicating higher pain burden. Mean

(SD) scores were 14.3 (23) for male survivors, 14.0 (20) for the male reference population ($p>0.05$), 19.7 (27) for female survivors and 20.8 (24) for the female reference population ($p>0.05$). General linear models – adjusted for age, tumour type, treatments received, cancer stage, number of comorbidities, the time between diagnosis and questionnaire, partner status, educational level, employment status – highlighted differences between male and female survivors for each cancer type. Mean (SD) scores for male and female colorectal survivors were 13.9 (23) and 19.7 (26) respectively ($p<0.01$), male and female haematologic survivors were 17.4 (26) and 22.9 (29) respectively ($p<0.05$) and male and female thyroid survivors were 13.8 (24) and 18.5 (25) respectively ($p>0.05$) [2].

In the US cross-sectional study by Sanford et al., participants aged 18 or over with (n=7,565) and without (n=107,526) cancer from the NHIS (2010-2017) were compared on their levels of chronic pain [8]. Cancer survivors were diagnosed ≥2 years before survey administration. The cancer group combined survivors of breast, prostate, melanoma, cervix, colorectal, haematologic, uterus, ovarian, bladder, lung, kidney, head and neck and sarcoma cancer. Chronic pain was defined as the experience of pain most or every day for the past three months. The prevalence of chronic pain was greater among those with a cancer diagnosis (30.8%), compared to those without (15.7%). There was a significant difference between the groups, with cancer survivors 1.48 times as likely as the non-cancer group to have chronic pain (aOR: 1.48; 95% CI: 1.38, 1.59; $p<0.001$). When stratified by cancer diagnosis, sarcoma survivors had the greatest odds of chronic pain (aOR: 2.62; 95% CI 1.67, 4.13) in comparison to non-cancer control. Only bladder, prostate and breast cancer survivors had a reduced odds of chronic pain compared to those without a history of cancer (no statistics provided, only a forest plot). Compared to 18-34-year-old cancer survivors, all older age groups of cancer survivors were significantly more likely to report chronic pain, with adjusted odds ratios of: 1.64 (95% CI: 1.06, 2.56; $p=0.03$) for 35-44-year-olds, 1.77 (95% CI: 1.19, 2.62; $p=0.005$) for 45-54-year-

olds, 2.02 (95% CI: 1.39, 2.93; p<0.001) for 55-64-year-olds, 1.60 (95% CI: 1.10, 2.33; p<0.01) for 65-74-year-olds and 1.61 (95% CI: 1.11, 2.33; p<0.01) for ≥75-year-olds. The model adjusted for baseline demographic and socioeconomic characteristics, including sex, smoking status, race, ethnicity, age, insurance status, marital status and physical activity frequency [8].

Older adult survivors of lung (n=57; mean age=62.4 years), colon (n=117; mean age=64.6 years) and prostate (n=104; mean age=69.5 years) cancer were compared in the US cross-sectional study by Schag et al [33]. Participants were recruited from a larger study investigating quality of life and rehabilitation problems in cancer patients. Fifty-eight percent of lung cancer survivors (mean time since diagnosis=3.4 years) reported frequent pain, while 30% of colon (mean time since diagnosis=3.2 years) and 24% of prostate (mean time since diagnosis=3.5 years) cancer survivors reported frequent pain. No other statistical analysis was provided [33].

In Shin et al., a South Korean cross-sectional study, 50-70-year-old urological (prostate n=63, kidney n=84, bladder n=69) cancer survivors who had undergone curative surgery at four university hospitals between 2011 and 2013 and who were cancer-free for at least one year after surgery, were compared with subjects without a history of cancer (n=1,176) aged 40-70 years, recruited from a national survey [9]. The EORTC QLQ-C30 was used to assess pain, with higher scores equating to a greater burden of pain. The adjusted mean (SE) for pain in the general population was 8.7 (SE: 0.5), prostate cancer 6.7 (SE: 2.2; p=0.803), kidney cancer 15.1 (SE: 1.9; p<0.05), and bladder cancer 10.8 (SE: 2.1; p=0.787). The mean was adjusted for age, sex, education, employment status, smoking and alcohol consumption co-variates [9].

In the US cross-sectional study by Warner et al., 2,025 non-skin cancer survivors were compared with 13,783 cancer-free individuals. Both groups were recruited from the 2010-2012 HRS and were aged 50 years or over [10]. Survivors reported a cancer diagnosis at least two years before the interview. A higher prevalence of severe pain was reported in cancer survivors

compared to cancer-free individuals (8.6% and 6.4% respectively; unadjusted $p<0.001$), using an undefined pain assessment method [10].

Zucca et al. – in their Australian cross-sectional study – investigated pain in 863 18-75-year-old cancer survivors five-six years post-diagnosis [19]. Participants were from the New South Wales (NSW) Central Cancer Registry. Breast (n=249), prostate (n=133), melanoma (n=131), colorectal (n=110), urogenital (other) (n=46), gynaecological (n=33), head and neck (n=32), lymphohematopoietic (n=50), thyroid/other endocrine (n=19), lung (n=15) and other (n=45) cancer survivors were included. The EORTC QLQ-C30 was used to assess pain. The percentage of survivors reporting experiencing pain 'quite a bit' or 'very much' was 21.4% for lung cancer, 10.2% for lymphohematopoietic cancer, 7.4% for breast cancer, 6.7% for colorectal cancer, 5.6% for prostate cancer, 1.7% for melanoma and 0% for thyroid cancer. There was no significant difference in pain between the cancer types ($p=0.132$) [19].

Pain – individual cancer type

Breast

There were 19 studies (12 cross-sectional, seven cohort studies) involving survivors of breast cancer [4, 13, 14, 40-55]; among them two incorporated a control/comparison group [13, 14].

In the US cross-sectional study by Edmond et al., 200 survivors (≥21 years of age) of stage I-III breast cancer, 6-15 months post-breast conserving surgery were compared with 150 women aged 40 or over without a history of breast cancer or breast surgery [13]. Across all four pain-related outcomes there was a statistically significant difference between the two groups. Forty-seven percent of survivors experienced persistent breast pain (≥6 months), compared to 13% of the control group ($p<0.001$). Twenty-nine percent of survivors experienced clinically significant persistent breast pain (intensity 3+/10, ≥6 months), compared to 5% of the control group ($p<0.001$). The BPI breast pain intensity score was 1.63 (SD: 1.73) for survivors and

0.37 (SD: 0.94) for the control group (p<0.001), with a higher score equating to greater pain. The average unpleasantness of breast pain in the past month using a 0-100 visual analogue scale (VAS) – with higher scores indicating greater pain – was 11.50 (SD: 17.07) for survivors and 2.77 (SD: 8.47) for the control group (p<0.001) [13].

In a Canadian cohort study by Hsu et al., 535 women with localised stage I-III breast cancer (on average 12.5 years post-diagnosis, mean (SD) age: 49.8 ± 8.9 years at diagnosis) were recruited between 1989 and 1996 (166 had longitudinal quality of life data at all time points), followed prospectively and compared with 167 age-matched women with no history of breast cancer presenting for mammograms (161 completed the questionnaire) [14]. Bodily pain was assessed using the Short-Form (SF-36) Health Survey. There was no significant difference between breast cancer survivors and the control group for bodily pain at long-term follow-up, with an adjusted mean score of 51.6 and 50.4 respectively (difference: -1.11; 95% CI: -2.94, 0.72) [14].

Colorectal

There were eight studies (six cross-sectional, two cohort) involving survivors of colorectal cancer [20, 56-62]; none incorporated a control/comparison group.

Prostate

There were two studies (one cross-sectional, one case-control) involving survivors of prostate cancer [15, 63]; one of which included a control/comparison group [15].

In the Dutch case-control study by van Stam et al., 644 prostate cancer survivors ≥5 years post-diagnosis of stage I-IV carcinoma in community hospitals were compared with an age-matched (55-85 years) sample of 644 prostate cancer-free men, recruited from general practices [15]. Bodily pain was assessed using the SF-36 and in this study the authors linearly transformed the scores to a 0 to 100 scale, with 100 indicating highest functioning or lowest level of bother.

The mean bodily pain score for the prostate cancer group was 79.5 (SD: 23.5), compared to 83.3 (SD: 22.8) for the control group ($p<0.01$; $\eta^2=0.01$) [15].

Thyroid

There were two studies (both cross-sectional) including survivors of thyroid cancer [16, 64]; one with a comparator group [16].

In a German cross-sectional study, 121 thyroid cancer survivors from an inpatient rehabilitation clinic were compared with 2,037 participants from the general population [16]. Pain was compared between the groups using the EORTC QLQ-C30. Participants with thyroid cancer had a mean pain score of 35.0 (SD: 33.6), whilst the control group had a mean score of 15.3 (SD: 24.4), with a higher score indicating a higher level of pain. The effect size for the comparison between the groups was 0.8 and the *B* coefficient of linear regression univariate analysis (mean difference between the groups) was -19.7 (95% CI: -25.7, -13.6) [16].

Leukaemia

There was one study in leukaemia survivors, a case-control study in Italy, including leukaemia survivors ≥18 years of age [17]. Cancer survivors were 244 acute promyelocytic leukaemia (APL) (a subtype of acute myeloid leukaemia) patients in complete remission and more than five years' post-diagnosis, who had previously participated in two large clinical trials. They were compared with 244 age- and sex-matched individuals from the Italian general population without cancer. Using the SF-36, there was no significant difference between the groups for mean bodily pain (APL: 80.1; control: 80.9; mean difference: -0.8 (95% CI: -5.3, 3.8); p=0.739) [17].

Lung

There was one study in lung cancer survivors; a cross-sectional study in Taiwan in 152 individuals, with no control group [65].

Melanoma

There was one study identified in melanoma survivors; a US cross-sectional study in participants recruited from the Skin Health Study (SHS), a population-based case-control study assessing the association between indoor tanning and the risk of melanoma [18]. Individuals aged 25-29 years diagnosed with invasive cutaneous melanoma between July 2004 and December 2007 were included and compared with a random sample, frequency-matched to cases 1:1 on age and sex. There were 724 melanoma survivors and 660 cancer-free individuals and the melanoma survivors were diagnosed on average 9.6 ± 1.0 years before survey completion. Using the SF-36, the mean (SD) body pain score for melanoma survivors was 76.9 ± 21.4, compared to 72.3 ± 21.7 for the control group (higher score equates to less pain). The age- and sex-adjusted mean difference was 4.67 (95% CI: 2.39, 6.95; $p<0.0001$). When using the full model – adjusting for age, sex, education, income, marital status, BMI category, smoking status and number of comorbidities – the mean difference was 3.89 (95% CI: 1.89, 5.89; $p=0.0001$; adj. p-value=0.01; Cohen's d=0.21) [18].

Multiple myeloma

There was one study identified in multiple myeloma survivors [66]. The cross-sectional study was conducted in the US in 239 survivors and did not include a comparator group.

Non-Hodgkin's lymphoma

One study in non-Hodgkin's lymphoma survivors was identified in the literature search [67]. The cohort study was conducted in South Korea in 370 survivors; however, it did not include a comparator group.

Kidney, oesophagus, uterus, bladder cancer

There were no studies on kidney, oesophagus, uterus, or bladder cancer identified.

Evidence on pain in relation to demographic and clinical characteristics in cancer survivors (age, time since diagnosis, cancer stage, treatment type and time since treatment)

In the cohort study by the ACTION Study Group, 5,249 first-time cancer survivors from eight low- and middle-income countries (Cambodia, Indonesia, Laos, Malaysia, Myanmar, Philippines, Thailand and Vietnam) were interviewed at baseline (within 12 weeks of diagnosis), three and 12 months after diagnosis [38]. Cancer sites included: mouth and pharynx (n=571); oesophagus (n=49); stomach (n=143); colon and rectum (n=552); liver (n=26); pancreas (n=26); trachea, bronchus and lung (n=226); melanoma (n=18); breast (n=1,654); cervix (n=598); uterus (n=127); ovary (n=123); prostate (n=27); bladder (n=20); lymphomas and multiple myeloma (n=241); leukaemia (n=195); and other malignant neoplasms (n=617). The EORTC QLQ-C30 was used to assess pain on a 0-100 scale, with a higher score indicating higher symptom burden. At 12 months post-diagnosis, mean (SD) pain increased with advancing age, 19.2 (24.1) for <45 years, 20.7 (25.7) for 45-54 years, 22.1 (24.9) for 55-64 years and 31.4 (28.3) for ≥65 years. Similarly, mean (SD) pain increased with cancer stage at diagnosis, 12.5 (18.0) for Stage I, 15.7 (21.1) for Stage II, 20.4 (26.1) for Stage III, 32.6 (32.0) for Stage IV and 21.1 (21.0) for haematological cancers (no stage). By treatment (categories not mutually exclusive as most patients received a combination), mean (SD) pain for surgery was 17.9 (24.5), 25.7 (25.8) for no surgery, 20.1 (24.9) for radiotherapy, 22.5 (25.8) for no radiotherapy, 21.7 (25.6) for chemotherapy and 20.2 (24.7) for no chemotherapy. The study also conducted multiple linear regression and logistic linear regression analyses for pain, with a standardised beta of 0.20 (95% CI: 0.12, 0.28) for age. Using Stage I as the reference, the Stage II standardised beta was 2.96 (95% CI: 0.07, 5.86), 6.32 (95% CI: 3.29, 9.36) for Stage III, 19.29 (95% CI: 15.91, 22.67) for Stage IV and 7.89 (95% CI: 3.99, 11.78) for haematological. Standardised betas for treatment were: surgery -4.81 (95% CI: -6.74, -2.88); radiotherapy 1.35 (95% CI: -0.39, 3.08); and chemotherapy -0.45 (95% CI: -2.45, 1.56) [38].

In the US cross-sectional study by Bao et al., 1,280 postmenopausal breast cancer survivors from the Wellness after Breast Cancer (WABC) trial who had previously taken or were currently taking aromatase inhibitors (AIs) were compared on several characteristics [41]. Participants responded to the question 'during the past 6 months, do you consider yourself to be a person who lives with chronic pain?' In multivariable logistic regression, cancer survivors aged ≤55 years were more likely to report chronic pain, compared to survivors >70 years (reference) (aOR: 1.69; 95% CI: 1.10, 2.60; p=0.016), whilst there was no difference between survivors aged 56-70 years and survivors >70 years (aOR: 0.96; 95% CI: 0.68, 1.35; p=0.80). There was no difference in chronic pain for groups diagnosed <5 years (reference), 5-10 years (aOR: 1.18; 95% CI: 0.83, 1.67; p=0.36) or >10 years (aOR: 0.99; 95% CI: 0.62, 1.58; p=0.98) prior, nor was there a difference for surgery type (lumpectomy (reference) vs. mastectomy; mastectomy aOR: 1.04; 95% CI: 0.74, 1.48; p=0.82), reconstructive surgery (yes vs. no (reference); yes aOR: 1.19; 95% CI: 0.81, 1.74; p=0.37) or duration of AI therapy (<1 year (reference), 1-3 years aOR: 1.09; 95% CI: 0.75, 1.58; p=0.66, >3 years aOR: 0.7; 95% CI: 0.47, 1.06; p=0.096) [41].

The Norwegian cross-sectional study by Bredal et al. identified factors independently associated with chronic pain after treatment in 832 breast cancer survivors treated two to six years before the start of the study [4]. Using the BPI, univariate analysis showed younger survivors had a higher odds of experiencing chronic pain (25-45 years OR: 3.52; 95% CI: 1.84, 6.73; p<0.0001; 46-55 years OR: 2.64; 95% CI: 1.5, 4.42; p<0.0001; 56-65 years OR: 1.36; 95% CI: 0.83, 2.24; p=0.26) compared to survivors aged 66-75 years. Chemotherapy (OR: 1.87; 95% CI: 1.25, 2.24; p<0.0001) and locoregional radiotherapy to the axillary level (OR: 1.69; 95% CI: 1.22, 2.34; p=0.001) were significant, while breast-conserving surgery (OR: 1.03; 95% CI: 0.77, 1.39; p=0.83), mastectomy (OR: 1.01; 95% CI: 0.76, 1.34; p=0.95), tamoxifen (OR: 1.03; 95% CI: 0.99, 1.72; p=0.06) and AIs (OR: 0.91; 95% CI: 0.68, 1.23;

p=0.54) were not. In the multivariable logistic regression model, there was a significant relationship between age and chronic pain (OR: 0.95; 95% CI: 0.93, 0.98; p<0.0001) [4].

In a retrospective questionnaire-based cross-sectional study in New Zealand, 201 breast cancer survivors who had undergone surgery between 2013 and 2016 were assessed on persistent pain after breast cancer surgery (PPBCS) [44]. PPBCS was defined as pain in the ipsilateral breast, arm, shoulder, axilla or chest wall daily six or more months after surgery. Multivariate logistic regression analysis of predictors for PPBCS 6-48 months after surgery showed that age (<50, 50–65, >65 years), radiation therapy (yes vs. no) and time since surgery (6–12, 12–24, >24 months) were not significant predictors. Reconstruction surgery (expander or implant prosthesis and autologous flap reconstruction) was the only significant predictor for PPBCS (aOR: 4.1; 95% CI: 1.3, 13.0; p=0.02) [44].

In the Irish cross-sectional study by Drury et al., 252 colorectal survivors between six months and five years post-diagnosis were investigated [56]. Pain and pain intensity were assessed using the EuroQOL and FACT-C items, with higher scores indicating greater pain. Pain remained stable between baseline (mean (SD) score: 0.5 (0.7)) and year 5 (0.4 (0.7)). There was a significant effect for age, with an odds ratio of 2.3 (p≤0.005) for >65-year-olds compared with ≤65-year-olds for 'pain today' in chi-square analysis. Those not receiving radiotherapy (OR: 2.2; p≤0.05) or chemotherapy (OR: 3.6; p≤0.005) were significantly more likely to report 'pain today' relative to those who received radiotherapy or chemotherapy respectively [56].

In the US cohort study by Given et al., 841 survivors (≥65 years) of breast (26%), colon (19%), lung (26%) and prostate (29%) cancer were compared on pain, answering the question 'during the past 2 weeks, as a result of your cancer or its treatment have you experienced pain?' [25]. The proportion of participants reporting pain slightly decreased over time – using time since diagnosis – with 12% reporting pain at 6-8 weeks, 11% at 12-16 weeks, 10% at 24-30 weeks

and 10% at 52 weeks. It should be noted that there was considerable loss to follow-up at each stage [25].

In the Israeli cross-sectional study by Hamood et al., 410 primary nonmetastatic invasive breast cancer survivors were compared on chronic pain for several characteristics [47]. The study used the International Association for the Study of Pain definition of 'persisting pain, either continuously or intermittently, for more than 3 months after treatment.' Multivariable analysis showed a significant effect for age at time of study (for each additional year) (OR: 0.96; 95% CI: 0.94, 0.99; p=0.002), type of surgery-mastectomy compared to breast-conserving surgery (reference) (mastectomy OR: 3.54; 95% CI: 1.46, 8.59; p=0.005), radiotherapy compared to no radiotherapy (reference) (radiotherapy OR: 2.96; 95% CI: 1.43, 6.12; p=0.003) and time since diagnosis (for each additional year) (OR: 0.82; 95% CI: 0.75, 0.90; p<0.001) [47].

The Danish study by Johannsen et al. investigated high pain frequency in a cohort (n=1,905) of 18-70-year-old invasive breast cancer survivors [48]. Pain was assessed at 15 months and 7-9 years post-surgery, with subjects asked how often pain was experienced at two body locations (arm/shoulder where operated and surgical area). High pain frequency was defined as pain 'almost every day' or more frequently. At 15 months, younger survivors were more likely (p<0.001) to report high pain frequency compared to older survivors in unadjusted analysis (<40 years OR: 2.09; 95% CI: 1.30, 3.38; 40-49 years OR: 1.68; 95% CI: 1.28, 2.19; 50-59 years OR: 1.65; 95% CI: 1.31, 2.07), with a reference group of 60-71 years. The relationship held (p<0.001) at 7-9 years post-diagnosis in the unadjusted analysis, however in analysis adjusted for pain at 15 months and age, there was no significant difference by age. There was no significant effect of tumour grade at 15 months or 7-9 years in the adjusted analysis (p=0.25 and p=0.92, respectively), nor was there for type of surgery (mastectomy or lumpectomy), chemotherapy or radiotherapy [48].

In a US cross-sectional study of ≥18-year-olds previously described, 1,904 cancer survivors and 29,092 cancer-free individuals were compared [7]. Data were from the 2002 NHIS and included breast (17.1% of sample), prostate (10.2%), colorectal (7.6%), cervix (9.3%), uterus (5.6%), melanoma (5.6%), multiple (9.5%) and other cancer survivors. Participants responded yes/no to the question 'during the past 12 months have you had recurring pain?' Using 0-1 year since diagnosis as the reference, the adjusted odds ratio for pain was 0.83 for 2-5 years since diagnosis (95% CI: 0.61, 1.13), 0.99 for 6-10 years (95% CI: 0.70, 1.40) and 1.06 for 11 years or greater (95% CI: 0.78, 1.45). The models were adjusted for age, education, race/ethnicity and income [7].

In the Danish cross-sectional study by Peuckmann et al., individuals who had survived for at least five years post-surgery for primary breast cancer, without recurrence (n=1,316) were compared on multiple characteristics [50]. Chronic pain was assessed by the question 'have you had pain of more than 6 months' duration?' Multiple logistic regression analyses were conducted with a stepwise elimination procedure, using variables with p<0.25 from the univariate analysis. Older breast cancer survivors were less likely (p=0.001) to report chronic pain compared to a control group of 40-49-year-old survivors (70+ years OR: 0.48; 95% CI: 0.30, 0.76; 60-69 years OR: 1.01; 95% CI: 0.72, 1.42; 50-59 years OR: 1.20; 95% CI: 0.89, 1.62). There was a significant difference for time since operation, with those 5-10 years post-operation more likely than >10 years to report chronic pain (OR: 1.35; 95% CI: 1.01, 1.81). There was no difference in chronic pain between those receiving and not receiving radiotherapy [50].

In the Spanish cohort study by Romero et al., 1,057 surgically treated breast cancer survivors were assessed on persistent pain, defined as pain in the area of the operated breast, axilla, shoulder or arm in some of the follow-up visits at least three months after surgery [53]. The prevalence of persistent pain was 11.8% for 50-54-year-olds, 13.2% for 55-59-year-olds,

10.5% for 60-64-year-olds and 8.7% for 65-70-year-olds (p=0.483). Persistent pain prevalence was 8.3% for Stage I, 13.2% for Stage II and 9.3% for Stage III survivors (p=0.101). The prevalence of persistent pain among those receiving chemotherapy was 14.5%, compared to 8.4% for those not receiving chemotherapy (p<0.01), whilst prevalence was 11.7% for those receiving radiotherapy and 9.7% for those not receiving radiotherapy (p=0.457). There was a similar prevalence for radical (12.1%) and conservative (11.1%) surgery (p=0.693). In multivariate logistic regression analysis there was a reduced odds of persistent pain in older survivors (compared to younger) and a greater odds in those receiving chemotherapy after surgery (compared to those who did not), however both of these results were not statistically significant [53].

The US cross-sectional study by Schneider et al. surveyed 474 adult survivors (18-80 years of age) of colon and rectal cancer who had been part of a larger study of the quality of care for cancer patients [62]. The survey was administered approximately four years post-diagnosis. Questions on pain were adapted from previous studies, with subjects asked how often during the past four weeks they had experienced pain, cramps, or abdominal discomfort. There was no difference in the adjusted proportion reporting pain/cramps/abdominal discomfort 'fairly often' or 'very often' in the past four weeks for those receiving chemotherapy, compared to not (p=0.96) and receiving radiotherapy compared to not (p=0.27) [62].

In the US cross-sectional study by Smith et al., pain outcomes among 2,487 survivors of either colon or breast cancer were compared [35]. The pain assessment tool was adapted from previously validated instruments used in US government-funded studies and the questions were similar to those in the CAHPS Cancer Care Survey. Compared to female breast cancer survivors (no radiation) (reference group), female breast cancer survivors (radiation) were significantly more likely to report pain (OR 1.54; 95% CI: 1.20, 1.98; p<0.001). Compared to female breast cancer survivors (no radiation), female colon survivors (no radiation) were significantly less likely to

report pain (OR: 0.47; 95% CI: 0.31, 0.71; p<0.001). Male colon cancer survivors (no radiation) were significantly less likely to report pain compared to female breast cancer survivors (no radiation) (OR: 0.49; 95% CI: 0.33, 0.73; p<0.001). There was a higher prevalence of pain in those 0-6 months since surgery (71.6%) compared to those >6 months (58.4%) (reference), with multilevel logistic regression showing a significant relationship (0-6 months OR: 1.70; 95% CI: 1.31, 2.20; p<0.001). Similarly, recent chemotherapy reported a higher prevalence of pain (74.2% for 0-6 months ago, OR: 2.39; 95% CI: 1.82, 3.12; p<0.001, 67.4% for >6 months ago, OR: 1.35; 95% CI: 0.96, 1.90; p>0.05, and 51.5% for never receiving chemotherapy (reference)). Younger survivors of cancer reported a higher prevalence of pain (76.2% for <55, 66.2% for 55-64, 48.7% for 65-74, 30.5% for ≥75 years). The relationship was significant in logistic regression (OR: 0.96; 95% CI: 0.95, 0.97; p<0.001). There was no difference in the odds for pain when comparing Stage II and Stage III cancer survivors with Stage I survivors [35].

In the Norwegian cross-sectional study by Solvik et al., the effect of age on pain outcomes in 174 older home-dwelling cancer survivors was investigated [36]. Cancer survivors of either breast (n=12), prostate (n=20), lymphoma (n=10), lung (n=14), colon (n=15), brain (n=3), rectal (n=9), bladder (n=5), ovarian (n=12), or other (n=21) cancer were included. There was no significant difference (p=0.34) in pain outcomes between younger (≥65 and ≤77 years) and older (>77 years) cancer survivors, using the Norwegian version of the Edmonton Symptom Assessment System (ESAS-r) [36].

Storey et al. conducted a cross-sectional study in 1,127 breast cancer survivors three-eight years post-diagnosis, aged either 45 years and younger ('younger survivors') or 55-70 years ('older survivors') [54]. Pain was measured as part of a broad assessment of chemotherapy-induced peripheral neuropathy symptoms, using the Symptom Survivor Checklist – higher scores for each of the 12 items indicated greater symptom distress. There was a significant difference

($p<0.001$) in pain between older and younger survivors, with younger survivors reporting a higher prevalence (37% vs. 25%). Logistic regression analysis – controlled for sociodemographic and medical variables – showed a similar result (OR older vs. younger: 0.623; 95% CI: 0.469, 0.827; $p=0.001$) [54].

The cohort study by Subramaniam et al. involved 1,490 newly diagnosed cancer patients from nine Malaysian states, recruited from the ACTION study described above [37]. Mouth (n=118), colorectal (n=181), breast (n=466), female reproductive (n=94), lymphoma (n=350) and other (n=281) cancer survivors were compared on pain by time since diagnosis, age and cancer stage at diagnosis. The EORTC QLQ-C30 was used to assess pain on a 0-100 scale, with a higher score indicating higher symptom burden. Study participants at baseline were within 12 weeks of a cancer diagnosis. Pain was highest at baseline (32.6, SD: 27.6) and decreased at three months (31.3, SD: 22.6) and 12 months (26.7, SD: 23.7) post-diagnosis. At 12 months post-diagnosis, the standardised beta from regression analyses (backward method) was 0.17 (95% CI: 0.05, 0.28; $p\leq0.05$) for age. Using early stage as the reference for the cancer stage at diagnosis comparison, the standardised beta was 12.57 (95% CI: 9.03, 16.10; $p\leq0.001$) for late stage and 10.07 (95% CI: 5.47, 14.68; $p\leq0.001$) for haematologic tumour [37].

Pain interference

There were four studies (three cross-sectional [3, 19, 22] and one cohort [58]) identified that assessed pain interference, one of which included a control/comparison group without cancer [3].

In Bøhn et al., a cross-sectional study in Norway, 1,088 cancer survivors of either breast (n=440), colorectal (n=120), non-Hodgkin's lymphoma (NHL) (n=172), acute lymphoblastic leukaemia (ALL) (n=110) or non-metastatic malignant melanoma (NMMM) (n=246) diagnosed at the age of 19-39 years, were compared on pain interference with normal work, using the 12-item Short Form Survey (SF-12) [22]. Mean (SD) time since diagnosis was 15.2

(6.8) years. Ten percent of the whole sample experienced pain interference, and by cancer type 11% each of breast, colorectal and NHL, 10% of ALL and 8% of NMMM survivors experienced pain interference [22].

In the US cross-sectional study by Gallicchio et al., data from the CLUE II cohort study were analysed, comparing 1,261 survivors of multiple cancers with 1,261 age- and sex-matched individuals without a history of cancer [3]. The multiple cancer group combined breast (n=307), prostate (n=266), colorectal (n=144), uterus (n=88), melanoma (n=93) and other (23 other cancer types) (n=363). Participants were asked 'compared to two years ago, does pain usually interfere with your normal work (including both work outside the home and housework)?' There was a statistically significant difference (p=0.004) between the groups with 17.7% of cancer survivors reporting pain interferes 'quite a bit/extremely', compared to 13.8% of the control group. For 'moderate' interference, 19.0% of cancer survivors and 18.2% of control agreed, and for 'not at all/a little bit' 61.7% of cancer survivors and 66.2% of the control group agreed [3].

In the US cohort survey by Kenzik et al., 2,961 newly diagnosed survivors of colorectal cancer were followed for one year (one-year post-diagnosis) to determine predictors of pain interference [58]. Pain interference – both work outside the home, and housework – was assessed using the Medical Outcomes Study Short-Form-12 (MOS SF-12), with higher scores corresponding to greater pain interference. The BPI was used in the assessment of pain severity with higher scores indicating greater severity. Average pain severity increased between baseline (mean (SD): 1.45 (2.29)) and follow-up (mean (SD): 3.80 (2.08)), and mean (SD) 'pain severity at its least' followed a similar pattern (0.76 (1.63) to 1.98 (2.06)). Factors significantly associated with new/continued/higher pain interference in logistic regression at follow-up were age (OR 65-74 years vs. <55 years: 0.46; 95% CI: 0.33, 0.63; OR ≥75 years vs. <55 years: 0.66; 95% CI: 0.47-0.93), being female (OR: 1.33; 95% CI: 1.07, 1.65),

chemotherapy and radiation (OR compared to neither: 1.78; 95% CI: 1.24, 2.55). There was no effect for being aged 55-64 years, stage at diagnosis, chemotherapy or radiation only [58].

Zucca et al. – in their Australian cross-sectional study, previously described – investigated pain in 863 18-75-year-old cancer survivors five-six years post-diagnosis [19]. Participants were from the NSW Central Cancer Registry. Breast (n=249), prostate (n=133), melanoma (n=131), colorectal (n=110), urogenital (other) (n=46), gynaecological (n=33), head and neck (n=32), lymphohematopoietic (n=50), thyroid/other endocrine (n=19), lung (n=15) and other (n=45) cancer survivors were included. The EORTC QLQ-C30 was used to assess pain. The percentage of survivors reporting pain interference 'quite a bit' or 'very much' was 4.6% for breast cancer, 3.2% for prostate cancer, 0.8% for melanoma cancer, 5.7% for colorectal cancer, 6.1% for lymphohematopoietic cancer, 0% for thyroid cancer and 14.3% for lung cancer. There was no significant difference in pain interference between the cancer types (p=0.081) [19].

Discussion

Whilst it is challenging to extrapolate definitive results from the review given the heterogeneous nature of the identified studies, there are some general observations which may be made. It should also be noted that many studies relied upon unadjusted analyses or descriptive statistics. In studies including individuals with multiple cancer diagnoses, unspecified diagnoses or grouping single diagnoses in one group, cancer survivors were more likely than individuals without cancer to report pain. Younger cancer survivors were more likely than older cancer survivors to report pain. The majority of studies assessing time since diagnosis found no difference in pain outcomes by time, with some reporting increased likelihood of pain when closer to diagnosis, and one showing pain with more time since diagnosis. Recent treatment (chemotherapy or surgery) was associated with increased reporting of pain, however there were very few studies assessing this factor.

Pain outcomes differed by cancer diagnosis, and in general, lung, kidney, and uterus cancer survivors were more likely to report pain compared to colorectal, thyroid, prostate, and melanoma cancer survivors who were less likely. Mixed findings were observed in survivors of breast cancer, bladder cancer, leukaemia, and non-Hodgkin's lymphoma, and there was insufficient data in survivors of multiple myeloma and oesophagus cancer to form a conclusion. There was no clear trend for pain outcomes by cancer stage.

Comparing cancer survivors who received radiotherapy with those who did not, most studies showed no difference in pain outcomes, some showed increased pain in those receiving radiotherapy, and two showed reduced pain outcomes in those receiving. In studies reporting chemotherapy treatment, the majority reported increased pain in those receiving chemotherapy compared to those not receiving. The majority of studies assessing cancer surgery showed no difference in pain outcomes for those receiving and those not receiving surgery.

There was limited data on pain interference, with only four identified studies on the outcome. In the one study including a control group, pain interference was statistically significantly greater in cancer survivors compared to those without cancer. There was no difference in pain interference for different cancer types, although only two studies included this measure. In the one cohort study assessing predictors of new/continued/higher pain interference, younger age and having both chemotherapy and radiotherapy (compared to neither) were associated with increased odds.

Research gaps

Gaps, relevant to our study, include:

1. Out of the 67 studies which met the inclusion criteria for this review, only 17 included a control/comparison group without cancer, with the number of cancer survivors ranging from 121 to 7,565; only eight studies had at least 1,000 cancer survivors.

2. Pain interference was only measured in four studies included this review, and only one of these included a control group without cancer.
3. There were only two studies (one cohort and one cross-sectional) published in Australia, and neither included a control/comparison group. A large proportion of studies (n=26) were published in the US.
4. A large proportion of studies (n=19) recruited survivors of only one cancer type (breast cancer). Very few studies were identified in survivors of kidney, oesophagus, leukaemia, and multiple myeloma cancers. Highly specific cancer types were included in some instances e.g. acute promyelocytic leukaemia.
5. Over half (n=39) of the 67 studies were published in 2017 or earlier. Given that recruitment/assessment often occurs many years before studies are published, this means there is a lack of recent data available-problematic considering rapid advances in treatment for cancer and pain.
6. Several studies investigated pain outcomes in cancer survivors who had received specific treatments/medications (e.g. thyroidectomy, mastectomy) which limits ability to be directly compared with our analysis.
7. Follow-up time varied, with some studies focusing on post-surgery pain and some conducting follow-up several years later, which make comparisons difficult.
8. In several studies, pain was only assessed at certain body parts or regions, e.g. breast pain several months following breast conserving surgery, head/neck, and low back pain. In one study, pain was grouped with cramping and abdominal discomfort.
9. Instruments, tools or scales used to assess pain differed between studies, making comparisons difficult. Some questions were vague e.g. 'are you often troubled with pain?' Pain in last 30 days has potential for recall bias. One study used pain 'signs' (e.g.

grimacing, grinding teeth) and didn’t involve a direct assessment or questionnaire. Some used unvalidated questionnaires.

References

1. Reyes-Gibby CC, Aday LA, Anderson KO, Mendoza TR, Cleeland CS. Pain, depression, and fatigue in community-dwelling adults with and without a history of cancer. J Pain Symptom Manage. 2006;32(2):118-28.
2. Oertelt-Prigione S, de Rooij BH, Mols F, Oerlemans S, Husson O, Schoormans D, et al. Sex-differences in symptoms and functioning in >5000 cancer survivors: results from the PROFILES registry. Eur J Cancer. 2021;156:24-34.
3. Gallicchio L, Kalesan B, Hoffman SC, Helzlsouer KJ. Non-cancer adverse health conditions and perceived health and function among cancer survivors participating in a community-based cohort study in Washington County, Maryland. J Cancer Surviv. 2008;2(1):12-9.
4. Schou Bredal I, Smeby NA, Ottesen S, Warncke T, Schlichting E. Chronic pain in breast cancer survivors: comparison of psychosocial, surgical, and medical characteristics between survivors with and without pain. J Pain Symptom Manage. 2014;48(5):852-62.
5. Andrykowski MA. Physical and mental health status of survivors of multiple cancer diagnoses: findings from the National Health Interview Survey. Cancer. 2012;118(14):3645-53.
6. Götze H, Taubenheim S, Dietz A, Lordick F, Mehnert A. Comorbid conditions and health-related quality of life in long-term cancer survivors-associations with demographic and medical characteristics. J Cancer Surviv. 2018;12(5):712-20.
7. Mao JJ, Armstrong K, Bowman MA, Xie SX, Kadakia R, Farrar JT. Symptom burden among cancer survivors: impact of age and comorbidity. J Am Board Fam Med. 2007;20(5):434-43.
8. Sanford NN, Sher DJ, Butler SS, Xu X, Ahn C, Aizer AA, et al. Prevalence of chronic pain among cancer survivors in the United States, 2010-2017. Cancer. 2019;125(23):4310-8.
9. Shin DW, Park HS, Lee SH, Jeon SH, Cho S, Kang SH, et al. Health-related quality of life, perceived social support, and depression in disease-free survivors who underwent curative surgery only for prostate, kidney and bladder cancer: comparison among survivors and with the general population. Cancer Res Treat. 2019;51(1):289-99.
10. Warner DF, Schiltz NK, Stange KC, Given CW, Owusu C, Berger NA, et al. Complex multimorbidity and health outcomes in older adult cancer survivors. Fam Med Community Health. 2017;5(2):129-38.
11. Richardson LC, Wingo PA, Zack MM, Zahran HS, King JB. Health-related quality of life in cancer survivors between ages 20 and 64 years: population-based estimates from the Behavioral Risk Factor Surveillance System. Cancer. 2008;112(6):1380-9.
12. Villani ER, Fusco D, Franza L, Onder G, Bernabei R, Colloca GF. Characteristics of patients with cancer in European long-term care facilities. Aging Clin Exp Res. 2022;34(3):671-8.
13. Edmond SN, Shelby RA, Keefe FJ, Fisher HM, Schmidt JE, Soo MS, et al. Persistent breast pain among women with histories of breast-conserving surgery for breast cancer compared with women without histories of breast surgery or cancer. Clin J Pain. 2017;33(1):51-6.
14. Hsu T, Ennis M, Hood N, Graham M, Goodwin PJ. Quality of life in long-term breast cancer survivors. J Clin Oncol. 2013;31(28):3540-8.
15. van Stam MA, van der Poel HG, Bosch J, Tillier CN, Horenblas S, Mols F, et al. Prevalence and correlates of mental health problems in prostate cancer survivors: a case-control study comparing survivors with general population peers. Urol Oncol. 2017;35(8):531.e1-.e7.
16. Singer S, Lincke T, Gamper E, Bhaskaran K, Schreiber S, Hinz A, et al. Quality of life in patients with thyroid cancer compared with the general population. Thyroid. 2012;22(2):117-24.
17. Efficace F, Breccia M, Avvisati G, Cottone F, Intermesoli T, Borlenghi E, et al. Health-related quality of life, symptom burden, and comorbidity in long-term survivors of acute promyelocytic leukemia. Leukemia. 2019;33(7):1598-607.
18. Vogel RI, Strayer LG, Engelman L, Nelson HH, Blaes AH, Anderson KE, et al. Comparison of quality of life among long-term melanoma survivors and non-melanoma controls: a cross-sectional study. Qual Life Res. 2017;26(7):1761-6.
19. Zucca AC, Boyes AW, Linden W, Girgis A. All's well that ends well? Quality of life and physical symptom clusters in long-term cancer survivors across cancer types. J Pain Symptom Manage. 2012;43(4):720-31.

20. Paul CL, Cameron E, Doran C, Hill D, Macrae F, Carey ML, et al. Experiences of colorectal cancer patients in the 2-years post-diagnosis and patient factors predicting poor outcome. Support Care Cancer. 2016;24(12):4921-8.
21. Alleaume C, Bendiane MK, Bouhnik AD, Rey D, Cortaredona S, Seror V, et al. Chronic neuropathic pain negatively associated with employment retention of cancer survivors: evidence from a national French survey. J Cancer Surviv. 2018;12(1):115-26.
22. Bøhn SH, Thorsen L, Kiserud CE, Fosså SD, Lie HC, Loge JH, et al. Chronic fatigue and associated factors among long-term survivors of cancers in young adulthood. Acta Oncol. 2019;58(5):753-62.
23. Erturk M, Yildirim Y, Kilic SP, Ozer S, Aykar FS. Pain and fatigue in elderly cancer patients: Turkey example. Holist Nurs Pract. 2015;29(3):167-73.
24. Gallaway MS, Townsend JS, Shelby D, Puckett MC. Pain among cancer survivors. Prev Chronic Dis. 2020;17(17):E54.
25. Given CW, Given B, Azzouz F, Kozachik S, Stommel M. Predictors of pain and fatigue in the year following diagnosis among elderly cancer patients. J Pain Symptom Manage. 2001;21(6):456-66.
26. Goncalves JP, Veiga D, Araujo A. Chronic pain, functionality and quality of life in cancer survivors. Br J Pain. 2020;15(4):401-10.
27. Green CR, Hart-Johnson T. Cancer pain: an age-based analysis. Pain Med. 2010;11(10):1525-36.
28. Green CR, Hart-Johnson T, Loeffler DR. Cancer-related chronic pain. Cancer. 2011;117(9):1994-2003.
29. Kjaer TK, Johansen C, Ibfelt E, Christensen J, Rottmann N, Høybye MT, et al. Impact of symptom burden on health related quality of life of cancer survivors in a Danish cancer rehabilitation program: a longitudinal study. Acta Oncol. 2011;50(2):223-32.
30. Moye J, June A, Martin LA, Gosian J, Herman LI, Naik AD. Pain is prevalent and persisting in cancer survivors: differential factors across age groups. J Geriatr Oncol. 2014;5(2):190-6.
31. Oh SY, Shin SW, Koh SJ, Bae SB, Chang H, Kim JH, et al. Multicenter, cross-sectional observational study of the impact of neuropathic pain on quality of life in cancer patients. Support Care Cancer. 2017;25(12):3759-67.
32. Sager ZS, Wachen JS, Naik AD, Moye J. Post-traumatic stress disorder symptoms from multiple stressors predict chronic pain in cancer survivors. J Palliat Med. 2020;23(9):1191-7.
33. Schag CAC, Ganz PA, Wing DS, Sim MS, Lee JJ. Quality of life in adult survivors of lung, colon and prostate cancer. Qual Life Res. 1994;3(2):127-41.
34. Schulz KH, Patra S, Spielmann H, Klapdor S, Schluter K, van Eckert S. Physical condition, nutritional status, fatigue, and quality of life in oncological out-patients. Sage Open Med. 2017;5:2050312117743674.
35. Smith TG, Troeschel AN, Castro KM, Arora NK, Stein K, Lipscomb J, et al. Perceptions of patients with breast and colon cancer of the management of cancer-related pain, fatigue, and emotional distress in community oncology. J Clin Oncol. 2019;37(19):1666-76.
36. Solvik E, Ytrehus S, Utne I, Grov EK. Pain, fatigue, anxiety and depression in older home-dwelling people with cancer. Nurs Open. 2020;7(1):430-8.
37. Subramaniam S, Kong YC, Chinna K, Kimman M, Ho YZ, Saat N, et al. Health-related quality of life and psychological distress among cancer survivors in a middle-income country. Psychooncology. 2018;27(9):2172-9.
38. ACTION Study Group. Health-related quality of life and psychological distress among cancer survivors in Southeast Asia: results from a longitudinal study in eight low- and middle-income countries. BMC Med. 2017;15(1):10.
39. Zoëga S, Fridriksdottir N, Sigurdardottir V, Gunnarsdottir S. Pain and other symptoms and their relationship to quality of life in cancer patients on opioids. Qual Life Res. 2013;22(6):1273-80.
40. Bao T, Basal C, Seluzicki C, Li SQ, Seidman AD, Mao JJ. Long-term chemotherapy-induced peripheral neuropathy among breast cancer survivors: prevalence, risk factors, and fall risk. Breast Cancer Res Treat. 2016;159(2):327-33.

41. Bao T, Seidman A, Li Q, Seluzicki C, Blinder V, Meghani SH, et al. Living with chronic pain: perceptions of breast cancer survivors. Breast Cancer Res Treat. 2018;169(1):133-40.
42. Berger A, Kumar G, Levan T, Meza J. Symptom clusters and quality of life over 1 year in breast cancer patients receiving adjuvant chemotherapy. Asia Pac J Oncol Nurs. 2020;7(2):134-40.
43. Calderon C, Carmona-Bayonas A, Hernandez R, Ghanem I, Castelo B, de Castro EM, et al. Effects of pessimism, depression, fatigue, and pain on functional health-related quality of life in patients with resected non-advanced breast cancer. Breast. 2019;44:108-12.
44. Chiang DLC, Rice DA, Helsby NA, Somogyi AA, Kluger MT. The prevalence, impact, and risk factors for persistent pain after breast cancer surgery in a New Zealand population. Pain Med. 2019;20(9):1803-14.
45. Costa WA, Monteiro MN, Queiroz JF, Goncalves AK. Pain and quality of life in breast cancer patients. Clinics. 2017;72(12):758-63.
46. Divella M, Vetrugno L, Bertozzi S, Seriau L, Cedolini C, Bove T. Patient-reported pain and other symptoms among breast cancer survivors: prevalence and risk factors. Tumori. 2020;106(6):480-90.
47. Hamood R, Hamood H, Merhasin I, Keinan-Boker L. Chronic pain and other symptoms among breast cancer survivors: prevalence, predictors, and effects on quality of life. Breast Cancer Res Treat. 2018;167(1):157-69.
48. Johannsen M, Christensen S, Zachariae R, Jensen AB. Socio-demographic, treatment-related, and health behavioral predictors of persistent pain 15 months and 7-9 years after surgery: a nationwide prospective study of women treated for primary breast cancer. Breast Cancer Res Treat. 2015;152(3):645-58.
49. Lam E, Wong G, Zhang LY, Drost L, Karam I, Yee C, et al. Self-reported pain in breast cancer patients receiving adjuvant radiotherapy. Support Care Cancer. 2021;29(1):155-67.
50. Peuckmann V, Ekholm O, Rasmussen NK, Groenvold M, Christiansen P, Møller S, et al. Chronic pain and other sequelae in long-term breast cancer survivors: nationwide survey in Denmark. Eur J Pain. 2009;13(5):478-85.
51. Rahool R, Haider G, Shahid A, Shaikh MR, Memon P, Pawan B, et al. Medical and psychosocial challenges associated with breast cancer survivorship. Cureus. 2021;13(2):e13211.
52. Rief W, Bardwell WA, Dimsdale JE, Natarajan L, Flatt SW, Pierce JP. Long-term course of pain in breast cancer survivors: a 4-year longitudinal study. Breast Cancer Res Treat. 2011;130(2):579-86.
53. Romero A, Tora-Rocamora I, Bare M, Barata T, Domingo L, Ferrer J, et al. Prevalence of persistent pain after breast cancer treatment by detection mode among participants in population-based screening programs. BMC Cancer. 2016;16(1):735.
54. Storey S, Cohee A, Von Ah D, Vachon E, Zanville NR, Monahan PO, et al. Presence and distress of chemotherapy-induced peripheral neuropathy symptoms in upper extremities of younger and older breast cancer survivors. J Patient Cent Res Rev. 2020;7(4):295-303.
55. Zomkowski K, Wittkopf P, Back BBH, Bergmann A, Dias M, Sperandio FF. Pain characteristics and quality of life of breast cancer survivors that return and do not return to work: an exploratory cross-sectional study. Disabil Rehabil. 2020;43(26):3821-6.
56. Drury A, Payne S, Brady AM. The cost of survival: an exploration of colorectal cancer survivors' experiences of pain. Acta Oncol. 2017;56(2):205-11.
57. Huang WD, Yang JJ, Liu Y, Liu CJ, Zhang X, Fu WQ, et al. Assessing health-related quality of life of patients with colorectal cancer using EQ-5D-5L: a cross-sectional study in Heilongjiang of China. BMJ Open. 2018;8(12):e022711.
58. Kenzik K, Pisu M, Johns SA, Baker T, Oster RA, Kvale E, et al. Unresolved pain interference among colorectal cancer survivors: implications for patient care and outcomes. Pain Med. 2015;16(7):1410-25.
59. Lowery AE, Starr T, Dhingra LK, Rogak L, Hamrick-Price JR, Farberov M, et al. Frequency, characteristics, and correlates of pain in a pilot study of colorectal cancer survivors 1-10 years post-treatment. Pain Med. 2013;14(11):1673-80.

60. Mols F, Beijers T, Lemmens V, Van Den Hurk CJ, Vreugdenhil G, Van De Poll-Franse LV. Chemotherapy-induced neuropathy and its association with quality of life among 2- to 11-year colorectal cancer survivors: results from the population-based PROFILES registry. J Clin Oncol. 2013;31(21):2699-707.
61. Mulder FECM, van Roekel EH, Bours MJL, Weijenberg MP, Evers SMAA. The burden of colorectal cancer survivors in the Netherlands: costs, utilities, and associated patient characteristics. J Cancer Surviv. 2021;16(5):1055–64.
62. Schneider EC, Malin JL, Kahn KL, Ko CY, Adams J, Epstein AM. Surviving colorectal cancer: patient-reported symptoms 4 years after diagnosis. Cancer. 2007;110(9):2075-82.
63. Gavin AT, Drummond FJ, Donnelly C, O'Leary E, Sharp L, Kinnear HR. Patient-reported 'ever had' and 'current' long-term physical symptoms after prostate cancer treatments. BJU Int. 2015;116(3):397-406.
64. Tagay S, Herpertz S, Langkafel M, Erim Y, Freudenberg L, Schopper N, et al. Health-related quality of life, anxiety and depression in thyroid cancer patients under short-term hypothyroidism and TSH-suppressive levothyroxine treatment. Eur J Endocrinol. 2005;153(6):755-63.
65. Liao YC, Liao WY, Shun SC, Yu CJ, Yang PC, Lai YH. Symptoms, psychological distress, and supportive care needs in lung cancer patients. Support Care Cancer. 2011;19(11):1743-51.
66. Richter J, Sanchez L, Biran N, Wang CK, Tanenbaum K, DeVincenzo V, et al. Prevalence and survival impact of self-reported symptom and psychological distress among patients with multiple myeloma. Clin Lymphoma Myeloma Leuk. 2021;21(3):e284-e9.
67. Kang D, Cho J, Kim IR, Kim MK, Kim WS, Kim SJ. Health-related quality of life in non-Hodgkin lymphoma survivors: a prospective cohort study. Cancer Res Treat. 2018;50(4):1051-63.
