# Supplementary material for: Pain and its interference with daily living in relation to cancer: a comparative population-based study of 16,053 cancer survivors and 106,345 people without cancer
Source: BMC Cancer. 2023 Sep 13;23:774. doi: 10.1186/s12885-023-11214-5 (PMC10498633; doi:10.1186/s12885-023-11214-5)
Supplement: Supplementary file 2 — Additional file 2: Table S1. ICD-10-AM codes for cancers included in the ‘other cancer’ group. Table S2. Clinical characteristics of cancer by cancer type. Table S3. Prevalence of bodily pain and high-impact pain by cancer type and time since cancer diagnosis. Table S4. Prevalence of bodily pain and high-impact pain by cancer type and recent treatment for cancer. Table S5. Prevalence of bodily pain and high-impact pain by cancer type and stage. Figure S1. Prevalence of bodily pain in cancer survivors versus individuals without cancer, in a range of population subgroups. [file 12885_2023_11214_MOESM2_ESM.pdf]

ADDITIONAL FILE 2: SUPPLEMENTARY TABLES AND FIGURES

Contents

Table S1. ICD-10-AM codes for cancers included in the ‘other cancer’ group 2
Table S2. Clinical characteristics of cancer by cancer type 3
Table S3. Prevalence of bodily pain and high-impact pain by cancer type and time since cancer diagnosis 4
Table S4. Prevalence of bodily pain and high-impact pain by cancer type and recent treatment for cancer 5
Table S5. Prevalence of bodily pain and high-impact pain by cancer type and stage 6
Figure S1. Prevalence of bodily pain in cancer survivors versus individuals without cancer, in a range of population subgroups 7

Table S1. ICD-10-AM codes for cancers included in the ‘other cancer’ group

ICD10 Code	Label	n
C00-C14	Malignant neoplasms of lip, oral cavity and pharynx	332
C15-C26	Malignant neoplasms of digestive organs C16, C17, C21-C26	292
C30-C39	Malignant neoplasms of respiratory and intrathoracic organs C30-C32, C37	83
C40-C41	Malignant neoplasms of bone and articular cartilage	16
C42	Vacant category to designate topography sites	4
C45-C49	Malignant neoplasms of mesothelial and soft tissue	93
C51-C58	Malignant neoplasms of female genital organs C51-C53, C56-C58	214
C60-C63	Malignant neoplasms of male genital organs C60, C62, C63	48
C64-C68	Malignant neoplasms of urinary tract C65, C66	37
C69-C72	Malignant neoplasms of eye, brain and other parts of central nervous system	79
C73-C75	Malignant neoplasms of thyroid and other endocrine glands C74, C75	10
C76-C80	Malignant neoplasms of ill-defined, secondary and unspecified sites C76, C80	76
C81-C96	Malignant neoplasms, stated or presumed to be primary, of lymphoid, haematopoietic and related tissue C81, C88, C90, C96	62
D37-D48	Neoplasms of uncertain or unknown behaviour	135
	Total	1481

ICD-10-AM: International Statistical Classification of Diseases and Related Health Problems, Tenth Revision, Australian Modification.

In analyses by cancer type, cancers were classified as breast (ICD-10-AM diagnosis code C50, women only), prostate (C61, men only), lung (C33–C34), melanoma (C43), colorectal (C18–C20), non-Hodgkin’s lymphoma (C82–C86), kidney (C64), oesophagus (C15), uterus (C54–C55, women only), bladder (C67), thyroid (C73), leukaemia (C91–C95), multiple myeloma (C90.0) and ‘other cancers’.

Table S2. Clinical characteristics of cancer by cancer type

	Prostate	Breast	Melanoma	Colorectal	NHL	Lung	Kidney	Uterus	Bladder	Leukaemia	Thyroid	Multiple myeloma	Oesophagus	Other cancer	Any cancer
**n**	4577	3118	2526	1847	607	272	327	350	206	270	284	130	58	1481	16053
**Median time since diagnosis (years)**	5.6	7.3	6.4	6.0	5.2	2.8	5.6	6.2	5.4	4.9	5.5	3.3	2.3	5.3	5.9
**Time since diagnosis (years)**, %															
<1	8.7	6.7	10.2	8.1	11.7	24.3	11.9	11.7	13.6	11.5	9.9	13.1	25.9	13.8	9.7
1 to <2	9.1	7.4	8.4	8.9	11.0	14.3	8.0	7.1	10.2	11.9	10.9	17.7	17.2	10.8	9.1
2 to <5	27.0	21.1	23.2	24.7	26.4	24.6	25.4	24.6	23.3	27.4	25.0	34.6	19.0	22.7	24.4
5 to <10	35.4	30.4	28.3	32.4	28.3	23.9	29.7	30.0	23.8	25.6	32.4	23.8	22.4	28.6	31.1
10 or more	19.8	34.3	30.0	25.8	22.6	12.9	25.1	26.6	29.1	23.7	21.8	10.8	15.5	24.1	25.7
**Stage**, %															
Localised to tissue of origin	58.4	59.2	90.0	43.2	N/A	54.0	76.8	69.4	51.0	N/A	70.8	N/A	55.2	37.5	56.8
Regional spread, adjacent organs and/or regional lymph nodes	10.7	34.7	4.8	44.3	N/A	25.0	13.5	19.7	15.0	N/A	15.5	N/A	20.7	22.5	19.4
Distant metastases	0.6	2.6	1.2	4.2	N/A	11.8	1.8	3.1	1.0	N/A	1.4	N/A	3.4	9.0	2.5
Unknown	30.3	3.5	4.0	8.3	N/A	9.2	8.0	7.7	33.0	N/A	12.3	N/A	20.7	31.0	21.2
**Treatment for any cancer in the past month**, %	13.9	22.2	8.0	14.3	19.6	29.8	11.3	12.3	21.8	21.9	9.5	56.9	25.9	18.6	16.0

Cancer staging is applicable to solid tumours only and is not relevant for non-Hodgkin's lymphoma (NHL), leukaemia, or multiple myeloma.

Percentages are out of column totals.

Breast and uterus cancer survivors include women only. There were 16 men with breast cancer. They are not reflected in breast cancer or 'other cancer' categories. Prostate cancer survivors include men only.

Diagnosis codes grouped under 'other cancers' and the corresponding numbers of participants, are included in Supplementary Table 1.

There were 1,260 participants without a record of cancer in the cancer registry, who self-reported that they received treatment for cancer in the last month; they have been retained in the 'no cancer' group and are not reflected in participants receiving cancer treatment.

Table S3. Prevalence of bodily pain and high-impact pain by cancer type and time since cancer diagnosis

Cancer type	Time since diagnosis <2 years				Time since diagnosis 2 or more years			
	Bodily pain		High-impact pain		Bodily pain		High-impact pain	
	% (n/N)	PR (95% CI)	% (n/N)	PR (95% CI)	% (n/N)	PR (95% CI)	% (n/N)	PR (95% CI)
Multiple myeloma	60.0 (24/40)	1.81 (1.40-2.33)	40 (16/40)	2.77 (1.89-4.07)	47.7 (42/88)	1.51 (1.21-1.88)	26.4 (23/87)	1.96 (1.37-2.79)
Lung	51.0 (51/100)	1.57 (1.29-1.91)	29.1 (30/103)	2.08 (1.53-2.83)	47.2 (76/161)	1.39 (1.18-1.64)	25.8 (41/159)	1.74 (1.33-2.27)
Leukaemia	41.9 (26/62)	1.28 (0.95-1.72)	19.7 (12/61)	1.38 (0.83-2.31)	40.2 (80/199)	1.27 (1.07-1.51)	18.7 (37/198)	1.38 (1.03-1.85)
Thyroid	35.6 (21/59)	1.10 (0.78-1.55)	--	--	42.9 (93/217)	1.32 (1.13-1.54)	24.9 (54/217)	1.83 (1.45-2.31)
Kidney	35.4 (23/65)	1.13 (0.81-1.56)	20 (13/65)	1.49 (0.92-2.43)	36.4 (90/247)	1.12 (0.95-1.33)	17.7 (44/248)	1.27 (0.97-1.66)
Uterus (women only)	48.4 (30/62)	1.35 (1.04-1.75)	29 (18/62)	1.87 (1.26-2.78)	39.5 (109/276)	1.08 (0.93-1.25)	21.1 (58/275)	1.32 (1.05-1.66)
Bladder	52.1 (25/48)	1.61 (1.22-2.13)	20.8 (10/48)	1.46 (0.82-2.60)	32 (48/150)	0.96 (0.76-1.21)	17.2 (26/151)	1.15 (0.81-1.63)
Breast (women only)	38.3 (165/431)	1.11 (0.98-1.25)	17.9 (77/431)	1.22 (1.00-1.50)	36.9 (958/2593)	1.05 (1.00-1.11)	15.6 (405/2601)	1.04 (0.95-1.14)
Prostate (men only)	28.4 (226/796)	0.99 (0.88-1.10)	11.1 (88/794)	0.92 (0.75-1.12)	31.9 (1168/3657)	1.07 (1.02-1.13)	13.7 (501/3653)	1.07 (0.98-1.17)
Oesophagus	41.7 (10/24)	1.30 (0.82-2.07)	--	--	--	--	--	--
Colorectal	31.9 (99/310)	0.95 (0.81-1.12)	15 (46/306)	1.02 (0.78-1.33)	35.7 (526/1473)	1.04 (0.97-1.12)	16 (235/1470)	1.06 (0.94-1.19)
Melanoma	32.9 (149/453)	1.03 (0.90-1.17)	14.9 (68/455)	1.09 (0.88-1.36)	32.7 (654/1999)	1.01 (0.95-1.07)	13.9 (277/1988)	1.00 (0.89-1.11)
NHL	33.3 (45/135)	1.04 (0.82-1.32)	18.4 (25/136)	1.33 (0.94-1.90)	33 (149/452)	0.99 (0.86-1.12)	16.7 (75/450)	1.15 (0.93-1.41)
Other cancer	44.3 (153/345)	1.35 (1.20-1.52)	24.3 (84/345)	1.71 (1.42-2.06)	35.4 (382/1080)	1.09 (1.01-1.19)	16.7 (180/1076)	1.21 (1.06-1.38)
Any cancer	35.7 (1048/2934)	1.12 (1.07-1.18)	17.1 (503/2933)	1.26 (1.16-1.37)	34.7 (4388/12636)	1.06 (1.04-1.09)	15.6 (1965/12617)	1.11 (1.06-1.16)
No cancer	31.3 (32471/103604)	1.00	13.1 (13573/103623)	1.00	31.3 (32471/103604)	1.00	13.1 (13573/103623)	1.00

CI: confidence interval; NHL: non-Hodgkin's lymphoma; PR: prevalence ratio.

Regression models estimated age- and sex- adjusted PRs for each cancer type and clinical characteristic separately. Clinical characteristics categories with at least 10 participants with the outcome of interest were included in the corresponding regression model.

Table S4. Prevalence of bodily pain and high-impact pain by cancer type and recent treatment for cancer

Cancer type	Recent treatment for cancer				No recent treatment for cancer			
	Bodily pain		High-impact pain		Bodily pain		High-impact pain	
	% (n/N)	PR (95% CI)	% (n/N)	PR (95% CI)	% (n/N)	PR (95% CI)	% (n/N)	PR (95% CI)
Multiple myeloma	61.6 (45/73)	1.93 (1.61-2.32)	39.7 (29/73)	2.91 (2.20-3.85)	38.2 (21/55)	1.18 (0.84-1.65)	18.5 (10/54)	1.32 (0.75-2.33)
Lung	55.8 (43/77)	1.75 (1.43-2.15)	34.6 (27/78)	2.53 (1.85-3.46)	45.4 (83/183)	1.33 (1.14-1.57)	24 (44/183)	1.62 (1.25-2.10)
Leukaemia	41.4 (24/58)	1.31 (0.96-1.79)	19 (11/58)	1.41 (0.82-2.44)	39.8 (80/201)	1.24 (1.05-1.47)	18.1 (36/199)	1.31 (0.98-1.77)
Thyroid	70.4 (19/27)	2.18 (1.68-2.83)	46.2 (12/26)	3.37 (2.18-5.21)	38.2 (95/249)	1.17 (1.00-1.37)	20.1 (50/249)	1.48 (1.15-1.90)
Kidney	45.9 (17/37)	1.43 (1.01-2.02)	16.2 (6/37)	--	34.2 (92/269)	1.06 (0.90-1.25)	18.2 (49/269)	1.31 (1.02-1.69)
Uterus (women only)	47.6 (20/42)	1.29 (0.94-1.76)	35.7 (15/42)	2.20 (1.46-3.30)	40.1 (118/294)	1.10 (0.96-1.27)	20.8 (61/293)	1.31 (1.05-1.64)
Bladder	48.8 (21/43)	1.46 (1.07-2.00)	27.9 (12/43)	1.88 (1.15-3.07)	33.8 (52/154)	1.02 (0.81-1.27)	15.5 (24/155)	1.04 (0.72-1.51)
Breast (women only)	44.4 (295/665)	1.29 (1.18-1.41)	20.7 (139/673)	1.42 (1.22-1.64)	35.1 (827/2356)	1.00 (0.94-1.06)	14.6 (343/2356)	0.97 (0.87-1.07)
Prostate (men only)	41.1 (255/621)	1.37 (1.25-1.51)	21 (131/623)	1.63 (1.40-1.91)	29.6 (1115/3770)	1.00 (0.95-1.05)	12 (450/3763)	0.95 (0.86-1.04)
Oesophagus	--	--	--	--	31.7 (13/41)	0.99 (0.63-1.57)	--	--
Colorectal	42.4 (109/257)	1.26 (1.09-1.46)	25.1 (64/255)	1.71 (1.38-2.11)	33.6 (508/1511)	0.98 (0.92-1.06)	14.3 (215/1506)	0.95 (0.84-1.07)
Melanoma	41.7 (80/192)	1.28 (1.08-1.52)	22.8 (44/193)	1.61 (1.24-2.09)	31.9 (716/2243)	0.99 (0.93-1.05)	13.3 (297/2234)	0.96 (0.86-1.06)
NHL	41.7 (48/115)	1.28 (1.03-1.59)	20.7 (24/116)	1.46 (1.02-2.09)	31 (146/471)	0.93 (0.81-1.07)	16.2 (76/469)	1.12 (0.92-1.38)
Other cancer	49.1 (131/267)	1.50 (1.33-1.70)	30.2 (81/268)	2.14 (1.78-2.58)	35 (401/1146)	1.08 (1.00-1.17)	16 (183/1141)	1.15 (1.01-1.32)
Any cancer	44.7 (1113/2490)	1.37 (1.31-1.43)	24 (600/2502)	1.71 (1.59-1.83)	33 (4271/12957)	1.01 (0.99-1.04)	14.3 (1849/12926)	1.02 (0.98-1.07)
No cancer	31.4 (32301/102927)	1	13.1 (13494/102948)	1	31.4 (32301/102927)	1	13.1 (13494/102948)	1

CI: confidence interval; NHL: non-Hodgkin's lymphoma; PR: prevalence ratio.

Regression models estimated age- and sex- adjusted PRs for each cancer type and clinical characteristic separately. Clinical characteristics categories with at least 10 participants with the outcome of interest were included in the corresponding regression model.

Table S5. Prevalence of bodily pain and high-impact pain by cancer type and stage

Cancer type	Localised/regional cancer				Distant metastasis			
	Bodily pain		High-impact pain		Bodily pain		High-impact pain	
	% (n/N)	PR (95% CI)	% (n/N)	PR (95% CI)	% (n/N)	PR (95% CI)	% (n/N)	PR (95% CI)
Lung	47.8 (99/207)	1.43 (1.24-1.66)	26.1 (54/207)	1.80 (1.42-2.27)	60 (18/30)	1.85 (1.36-2.52)	35.5 (11/31)	2.55 (1.54-4.22)
Thyroid	41.4 (98/237)	1.28 (1.10-1.49)	23.2 (55/237)	1.71 (1.36-2.17)	--	--	75 (3/4)	--
Kidney	34.4 (97/282)	1.07 (0.91-1.25)	17 (48/283)	1.22 (0.94-1.58)	--	--	33.3 (2/6)	--
Uterus (women only)	42.7 (129/302)	1.17 (1.03-1.34)	23.3 (70/301)	1.46 (1.19-1.80)	18.2 (2/11)	--	27.3 (3/11)	--
Bladder	35.7 (46/129)	1.08 (0.86-1.36)	15.3 (20/131)	1.04 (0.69-1.56)	--	--	--	--
Breast (women only)	36.9 (1048/2842)	1.06 (1.01-1.11)	15.5 (442/2851)	1.04 (0.95-1.14)	48.7 (37/76)	1.41 (1.12-1.79)	26 (20/77)	1.77 (1.21-2.59)
Prostate (men only)	30.4 (937/3084)	1.04 (0.98-1.10)	12.6 (390/3084)	1.01 (0.92-1.12)	46.2 (12/26)	1.52 (1.01-2.28)	8.3 (2/24)	--
Oesophagus	32.6 (14/43)	1.01 (0.65-1.56)	--	--	--	--	--	--
Colorectal	35.7 (557/1560)	1.04 (0.98-1.12)	16.2 (252/1553)	1.07 (0.96-1.20)	32 (24/75)	0.98 (0.70-1.37)	17.6 (13/74)	1.25 (0.76-2.05)
Melanoma	32.7 (761/2324)	1.01 (0.95-1.07)	14.2 (329/2314)	1.02 (0.92-1.13)	33.3 (10/30)	1.02 (0.61-1.72)	19.4 (6/31)	--
Other cancer	36.4 (312/858)	1.13 (1.03-1.23)	17.2 (147/854)	1.25 (1.08-1.45)	43 (55/128)	1.31 (1.08-1.60)	19.7 (25/127)	1.41 (0.99-2.00)
Any cancer	34.5 (4103/11882)	1.06 (1.03-1.09)	15.3 (1820/11873)	1.10 (1.05-1.15)	41.9 (164/391)	1.27 (1.13-1.43)	21.8 (85/390)	1.54 (1.28-1.87)
No cancer	31.3 (32471/103604)	1	13.1 (13573/103623)	1	31.3 (32471/103604)	1	13.1 (13573/103623)	1

CI: confidence interval; PR: prevalence ratio.

Cancer staging is applicable to solid tumours only and is not relevant for non-Hodgkin's lymphoma, leukaemia, or multiple myeloma.

Regression models estimated age- and sex- adjusted PRs for each cancer type and clinical characteristic separately. Clinical characteristics categories with at least 10 participants with the outcome of interest were included in the corresponding regression model.

Figure S1. Prevalence of bodily pain in cancer survivors versus individuals without cancer, in a range of population subgroups

	Without cancer % (n/N)	With cancer % (n/N)	PR (95% CI) of pain	p-value for interaction
**Overall**	31.3 (32471/103604)	34.9 (5436/15570)	1.07 (1.05-1.10)	
**Age group**				
45-64	28.2 (14924/52845)	32.4 (1405/4335)	1.15 (1.10-1.20)	<.0001
65-79	33.3 (13598/40774)	35.4 (2940/8306)	1.10 (1.06-1.13)	
≥80	39.5 (3949/9985)	37.2 (1091/2929)	1.00 (0.95-1.06)	
**Sex**				
Male	28.1 (12634/44940)	32.3 (2812/8704)	1.10 (1.06-1.14)	0.5875
Female	33.8 (19837/58664)	38.2 (2624/6866)	1.07 (1.04-1.10)	
**Region of residence**				
Major cities	30.0 (14866/49556)	33.1 (2510/7585)	1.06 (1.02-1.09)	0.3525
Inner regional	32.9 (11253/34245)	36.8 (1908/5191)	1.08 (1.04-1.13)	
Outer regional/remote/very remote	34.9 (3344/9570)	39.3 (551/1402)	1.11 (1.03-1.19)	
**Country of birth**				
Australian born	31.8 (25580/80452)	35.6 (4440/12467)	1.08 (1.05-1.11)	0.2684
Not Australian born	29.6 (6678/22586)	31.9 (954/2994)	1.03 (0.98-1.09)	
**Education**				
No school certificate	47.0 (3780/8038)	46.1 (663/1437)	1.00 (0.94-1.06)	0.0003
Certificate/diploma/trade	33.6 (21551/64223)	36.9 (3687/9995)	1.07 (1.04-1.10)	
Tertiary	22.3 (6789/30416)	25.9 (1027/3968)	1.11 (1.05-1.18)	

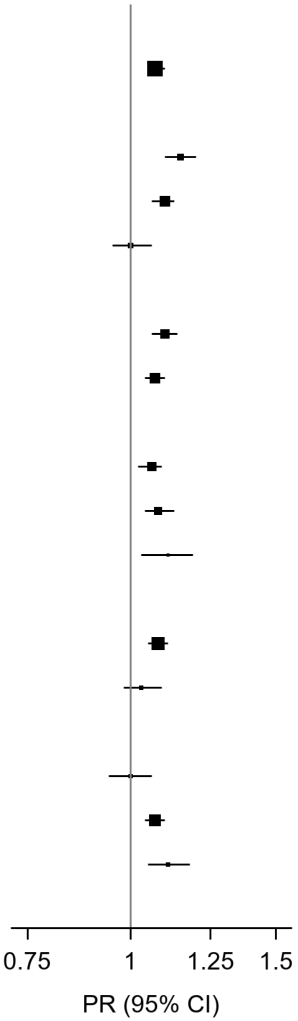

CI: confidence interval; PR: prevalence ratio.
